# Platelets Are at the Nexus of Vascular Diseases

**DOI:** 10.3389/fcvm.2019.00132

**Published:** 2019-09-11

**Authors:** Héloïse Lebas, Katia Yahiaoui, Raphaël Martos, Yacine Boulaftali

**Affiliations:** Laboratory of Vascular Translational Science, U1148 Institut National de la Santé et de la Recherche Médicale (INSERM), Sorbonne Paris Cite, Univ Paris Diderot, Paris, France

**Keywords:** vascular diseases, leukocytes, inflammation, platelets activation, thrombosis

## Abstract

Platelets are important actors of cardiovascular diseases (CVD). Current antiplatelet drugs that inhibit platelet aggregation have been shown to be effective in CVD treatment. However, the management of bleeding complications is still an issue in vascular diseases. While platelets can act individually, they interact with vascular cells and leukocytes at sites of vascular injury and inflammation. The main goal remains to better understand platelet mechanisms in thrombo-inflammatory diseases and provide new lines of safe treatments. Beyond their role in hemostasis and thrombosis, recent studies have reported the role of several aspects of platelet functions in CVD progression. In this review, we will provide a comprehensive overview of platelet mechanisms involved in several vascular diseases.

Since their first description by Osler (1) and later by Bizzozero (2), platelets have gained a lot of attention in many biological processes. Among the earliest evidence that platelets are crucial for human hemostasis, is based on platelet transfusions in thrombocytopenic patients that can restore hemostatic function. Platelets play a critical role in hemostasis by maintaining the integrity of blood vessels. They provide the first line of defense following injury, forming thrombi that patch-up damaged endothelial tissue and thereby play an indispensable role in hemostasis. However, dysregulated platelet activation can lead to thrombosis, myocardial infarction and stroke. Platelets are also involved in the development of atherosclerosis in coronary or carotid arteries, which is commonly the trigger for thrombosis. Plaque rupture is a common cause of arterial thrombosis and leads to the exposure of thrombogenic components to the flowing blood. The current dogma is that arterial thrombi are composed of aggregated platelets and venous thrombi are enriched in fibrin. However, this view has been challenged with landmark works by several groups on the role of platelets in venous thrombosis (3, 4). The molecular mechanism of thrombus formation has been extensively reviewed in detail elsewhere (5). Here we will provide a brief description of the role of platelets in clot formation and discuss their implication in vascular diseases.

## Platelet Mechanisms in Arterial Thrombosis and Vascular Inflammation

At sites of vascular injury, the subendothelial extracellular matrix (ECM) is exposed to the blood, to which platelets promptly adhere in order to limit hemorrhage and promote tissue healing. This matrix contains several adhesive macromolecules such as collagen, von Willebrand factor (vWF), laminin, fibronectin and thrombospondin, all of which serve as ligands for different platelet surface receptors. Among these subendothelial substrates, the thrombogenic fibrillar collagens type I and III are by far the most potent mediators of platelet adhesion due to their strong platelet activating potential and affinity for vWF. Transient adhesion of platelets (tethering) depends largely on vWF and its receptor, the GPIb-V-IX complex. Platelets express multiple surface receptors that directly or indirectly interact with collagen, among which integrin α2β1 (GPIa/IIa) and the Ig-like receptor glycoprotein VI (GPVI) are the most important ones. Integrin α2β1 predominantly mediates adhesion, whereas GPVI is the collagen-activated receptor in platelets. GPVI is a transmembrane protein of 62 kDa and belongs to the family of immunoreceptor tyrosine-based activation motif (ITAM) receptors. GPVI binds to the Fc receptor γ chain (FcRγ) which triggers the signaling cascade. Activation of platelets by GPVI–collagen interactions leads to the activation of phospholipase C and the subsequent mobilization of the second messengers calcium (Ca^2+^) and diacylglycerol (DAG). DAG is critical for protein kinase C (PKC) activation, a key event in platelet granule release and integrin activation (6). Ca^2+^ regulates various adhesive platelet responses such as integrin activation and the release of ADP and thromboxane A2 (TxA2) that can activate the G protein-coupled receptors (GPCRs), ADP purinergic receptors P2Y1 (7) and P2Y12 (8) and the thromboxane receptors (TP) TPα and TPβ. These second wave mediators allow the recruitment of circulating platelets which reinforce thrombus formation on collagen surfaces (9). In the clinic, pharmacological inhibition of TxA2 generation and/or the P2Y12 receptor are effective strategies to reduce thrombus formation at sites of vascular injury (10). Importantly, GPVI plays a central role in collagen-induced exposure of procoagulant phospholipids at the platelet surface, allowing efficient thrombin generation (11) and platelet activation. Of note, human platelets express the thrombin receptors, PAR1 and PAR4, whereas mouse platelets express a PAR3/PAR4 complex (12).

The specificity of GPVI is not restricted to collagen. Laminin as well as fibronectin, present in the basement membrane, have been shown to support platelet adhesion and spreading through α6β1 and GPVI (13, 14). The exclusive expression of GPVI on platelets makes it an attractive target. A recent placebo-controlled phase 1 study evaluated the safety and tolerability of a humanized Fab anti GPVI (Act017) in healthy donors. This study reported no bleeding events or increased of the bleeding time suggesting a promising effect of targeting GPVI in thrombotic diseases (15). The hemITAM receptor, C-type lectin 2 (CLEC2), may also contribute to platelet activation and thrombus formation in the deeper layers of the ECM. The known ligand for CLEC-2 is podoplanin which is expressed by type-1 alveolar cells, fibrotic reticular cells, lymphatic endothelial cells (EC) but not by vascular ECs. While the role of CLEC-2 in arterial thrombosis is still not clear (16–18), a recent *in vitro* study shows that podoplanin-expressing perivascular mesenchymal stromal cells are able to protrude through ECs and activate platelets in a CLEC-2 dependent manner (19). However, further *in vivo* studies are needed to support this observation in the context of inflammation or vascular injury.

Platelets are also known to play an important role in inflammation by recruiting leukocytes. This crosstalk has been well-studied and contributes to the increased leukocyte infiltration in tissue. The molecular mechanism of this cross-talk has been experimentally documented in different organs and in various inflammatory situations. Experimental studies show that thrombocytopenic animals have a significant reduction in leukocyte numbers in inflamed organs (20–22). Similarly, mice deficient for the main platelet adhesion receptors (P-selectin, GPIbα, GPVI, β3 integrin) show reduced tissue inflammation (23–25). Importantly, the direct interaction between platelets and leukocytes not only occurs locally but also in circulation. Increased levels of neutrophil/platelet and/or monocyte/platelet aggregates have been reported in patients with various inflammatory diseases (26–28). Platelet activation and secretion have been shown to recruit leukocytes, upregulate adhesion molecules by EC and destabilize EC junctions. Platelets can release a variety of chemokines (e.g., platelet factor 4, IL1β, PAF, RANTES) that can up regulate endothelial adhesion molecules (ICAM, αvβ3) (29), as well as the release of Weibel palade content and open endothelial junctions (30). Among the pro-permeable platelet factors, the GPVI-dependent serotonin release has been shown to contribute to the inflammation in the joints of arthritic mice (31). Furthermore, the release of soluble factors by platelets is also central to stimulate leukocytes. For example, platelet–released adenine nucleotides or platelet factor 4 and PDGF can favor superoxide anion generation by neutrophils (32). Conversely, activated neutrophils through leukocyte-released substances, such as platelet-activating factor (PAF), elastase, and cathepsin G, may induce platelet aggregation and secretion (33). The overall effect of the platelet-leukocytes interaction on tissue integrity can be either beneficial or deleterious depending of the inflammatory situation. This dichotomous aspect of the platelet-leukocyte interaction is further documented by the formation of neutrophil extracellular traps (NETs) that entrap bacteria (34) but also cause significant damage to the surrounding tissue (35). Based on the intertwined interaction between platelet and leukocyte in pathological situations, platelet/leukocyte aggregates (PLA) are rather important regulators of disease than just a read-out for platelet activation. The use of platelet inhibitors in patients with cardiovascular disease (e.g., clopidogrel, aspirin, eptifabitide) has been shown to reduce leukocyte recruitment, cytokine release and subsequently improve disease outcome. For instance, clopidogrel pretreatment in addition of ASA therapy was shown to reduce the C-Reactive protein (CRP) level, an inflammatory marker, in patients with percutaneous coronary intervention (36). Apart from antiplatelet therapy, anti-inflammatory agent such as colchicine has been used in various settings of cardiovascular disease (e.g., myocardial infarction) by inhibiting interleukin- 1 production by neutrophils (37, 38). Considering the anti-inflammatory properties of antiplatelet therapy, one could speculate that antiplatelet therapy could be considered as either a complementary or a second line of treatment to inflammatory medications (e.g., colchicine) in vascular disease.

## Role of Platelets in Early and Late Stages of Atherosclerosis

Atherosclerosis is a chronic inflammatory vascular disease involving ECs, vascular smooth muscle cells and mononuclear cells. Atherosclerosis classification as an inflammatory disease is based on the finding that immune competent cells and pro-inflammatory cytokines are abundant in atherosclerotic lesions. It is characterized by the formation of an atheromatous plaque mainly composed of pro-inflammatory oxidized low density lipoproteins (oxLDL) and foam cells accumulation in the intima of medium or large arteries, in high-shear stress areas. It results in vessel occlusion inducing CVD onset (39). The role of platelets in early atherosclerosis have been proposed by pioneer studies. Russel Ross postulated in his “*response to injury theory*” that “*lesions of atherosclerosis result from injury to the artery wall*” and result in “*subtle arterial endothelial cells desquamation*” (40). Of the many possible injuries, mechanical stresses may occur at particular anatomic sites and lead to the detachment of ECs from the artery wall and subsequently platelet adherence (41). Recent studies also reported endothelial breaches in the intima of human coronary arteries as well as in ApoE mice at sites of flow perturbation leading to the infiltration of red blood cells and leukocytes (42). Almost 30 years after Russel Ross hypothesis, the Massberg group in a landmark paper showed that in high-fat diet fed ApoE mice, platelet adhesion to the endothelium precedes the development of atherosclerotic lesions and leukocyte recruitment in atherosclerotic plaque supporting a major role of platelets in atherogenesis (43). Mechanistic studies showed that prolonged blockade of platelet adhesion in atherosclerosis animal model reduces leucocyte recruitment in arterial wall and results in fewer lesion formation (43). To decipher the involvement of platelets in this disease, several genetically modified mice lacking diverse platelet receptors were used in animal models of atherosclerosis.

Platelet glycoprotein Ibα (GPIbα), the ligand-binding subunit of the GPIb-V-IX receptor complex is known to interact with several proteins like vWF, P-selectin, Mac-1 and α-thrombin (44–47). Injections of anti-GPIbα antibodies in 10 weeks old ApoE^−/−^ mice reduced both platelet transient and firm adhesion to the vascular surface of the common carotid. Genetic depletion of the GPIbα subunit leads to severe thrombocytopenia and reduced atherosclerosis progression with smaller lesion area (48). This reported protective effect could be a consequence of thrombocytopenia since mice with extracytoplasmic GPIbα domain genetic deletion (IL4R/GPIbα mice) develop milder thrombocytopenia and are not protected against atherosclerosis (48). It indicates that GPIbα binding site for vWF, P-selectin, Mac-1 and α-thrombin might be dispensable for atherosclerosis development. This is quite surprising since vWF genetic depletion is protective in an animal model of atherosclerosis (49). Similarly, a role for Mac1/GPIbα interaction has been shown in leukocyte recruitment at sites of vascular inflammation (50, 51). The subunit GPIbβ has been also investigated in atherosclerosis by using GPIbβ^−/−^/ApoE^−/−^ mice fed a chow diet for 30 weeks. Despite the moderate thrombocytopenia of those mice, GPIbβ was found dispensable in atheroprogression (52).

Overall, these studies suggest that redundant mechanisms in platelet recruitment occur at site of developing atherosclerosis. The integrin αIIbβ3 can also mediate platelet adhesion via vWF binding, especially in modest shear stress condition as in large arteries. GPIIb genetic depletion results in reduced platelet adhesion at sites of vascular injury, decreased inflammatory processes and fewer atherosclerotic plaque formation (53). Integrin αIIbβ3 activation can be induced by GPIb-V-IX receptor, but also by GPVI receptor (54). Several GPVI inhibition strategies led to reduced platelet adhesion and attenuated atherosclerosis in ApoE^−/−^ mice (55).

Aside from platelet adhesion, platelet activation plays a significant role in atherogenesis. The presence of activated platelets was reported in the blood obtained from patients with unstable atherosclerotic disease (56). Increased platelet reactivity has been suggested as a potential mechanism contributing to the accelerated atherosclerosis seen in diabetic patients, via capillary microembolization and acute arterial thrombosis (57). Likewise, circulating activated platelets are involved in the formation of atherosclerotic lesions in ApoE^−/−^ mice (58). Those activated platelets interact with the atherosclerotic endothelium, leading to the delivery of pro-inflammatory chemokines (e.g., CCL5 and CXCL4) promoting adhesion molecule expression (58). PLA formation is required for neutrophil recruitment to inflamed tissues as animal models studies revealed that platelets activate neutrophils for an efficient adhesion to vascular endothelium via integrin up-regulation (59). Platelets can also bind to the inflamed endothelium, enhancing leukocytes adhesion to the vessel wall (60–62). Indeed, deletion of P-selectin, a marker of granule secretion, in platelets and/or ECs leads to significantly impaired early atherosclerotic lesion development in mice (63, 64). In addition, several studies addressing the contribution of platelet receptors have been conducted. The role of the ADP receptor P2Y12 has been extensively studied throughout the years. It has been shown that P2Y12 genetic depletion is protective in ApoE^−/−^ mice and mediates a reduced lesion area, an increased fibrous content at the plaque site and less inflammatory cells infiltration (65). However, the role of P2Y12 expressed by vascular smooth muscle cells cannot be excluded (66). Mice deficient for P2Y12 specifically in hematopoietic cells were generated, and a reduced atherosclerotic lesion formation was also reported (67). In contrast, treatment of ApoE^−/−^ mice with the P2Y12 inhibitor clopidogrel bisulfate was associated with inconsistent results. Clopidogrel administration induced delayed atherogenesis, a reduced lesion size, slower progression of atherosclerotic lesion (68–70). However, another study reported that clopidogrel-treated mice have the same atherosclerotic burden as control mice (70). Moreover, clopidogrel administration in mice with established atherosclerotic lesions show no longer beneficial effect (69). A more recent P2Y12 inhibitor, ticagrelor, has been tested in atherosclerosis models. Several studies conclude to a beneficial effect of ticagrelor administration, reporting a reduced lesion area and slower atherosclerotic lesion progression (71, 72). Nevertheless, one study related no effect on atherosclerotic lesion size in ticagrelor-treated mice, but showed an increased fibrous cap area along with a diminished ratio necrotic core/lesion area, indicating plaque stabilization process (73). Pharmacological inhibition is more likely to give variable results than a genetic approach. The differences in the experimental conditions and the inhibitor dose may be responsible for the discrepant results. The impact of the platelet thrombin receptor, PAR4, has been also investigated in atherosclerosis. Indeed, transfusion of thrombin-activated platelets into mice increases plaque formation, suggesting that thrombin-induced platelet activation might contribute to platelet-dependent atherosclerosis (58). However, PAR4 deletion is not protective in ApoE^−/−^ mice (74) suggesting other platelet activators than thrombin are involved.

Upon activation, platelets release soluble factors (e.g., PF4, CD40L, RANTES, and TXA2) enhancing their activation and leukocyte recruitment. Disruption of this amplification process leads to diminished atherosclerotic lesion formation. Indeed, PF4 or CD40L genetic deletion protects ApoE^−/−^ mice from atherosclerosis (75, 76). Inhibition of RANTES or its receptors alters the progression of an established atherosclerotic lesion (77, 78). Biological response modulators such as CD40L and its receptor CD40 have been shown to exacerbate atherosclerosis progression by promoting leucocyte recruitment via molecule adhesion expression in vascular ECs (79).

Platelet TXA2 generation is the product of cyclooxygenase-1 (COX-1) activation and contributes to the platelet activation amplification loop. The TXA2 receptor (TP) antagonist administration induces a slight reduction of atherogenesis (80), and TP deficient ApoE^−/−^ mice showed delayed lesion development and reduced atherogenesis compared to control (81). Disruption of COX-1 expression in ApoE^−/−^ mice induces a decrease in atherosclerotic lesion formation, attesting TXA2 deleterious role in this pathology (82). Acetylsalicylic acid, also known as aspirin, is one of the most widespread antiplatelet treatment and displays also anti-inflammatory properties. This irreversible COX-1 inhibitor blocks the formation of TXA2 in platelet, producing an inhibitory effect on platelet aggregation. Most animal studies reported a beneficial effect of low-dose aspirin administration in ApoE^−/−^ mice. Atherogenesis and lesion progression is reduced in aspirin-treated mice compared to control (83–85). Low-dose aspirin also delayed the progression of established and advanced vascular atherosclerotic lesions (86). However, some studies reported no effect of aspirin in ApoE^−/−^ mice (70, 80), and one described a deleterious long-term effect on atherosclerotic lesion progression (87). Overall, these studies suggest a functional hierarchy and redundancy between the different receptors in the role of platelets in atheroprogression.

Downstream of the platelet receptors, the signaling molecule GTPase Rap1 is a critical node in platelet response. The calcium and diacylglycerol-regulated guanine nucleotide exchange factor I (CalDAG-GEFI; RasGRP2) has been identified as the major calcium sensor in platelets regulating the Rap1 activation (88). Studies led by the Bergmeier group uncovered key roles of CalDAG-GEFI in platelet responses: integrin activation, platelet adhesion and secretion, TXA2 generation (89, 90). In an animal model of atherosclerosis, mice lacking CalDAG-GEFI specifically in hematopoietic cells have smaller lesions, reduced atherogenesis and decreased inflammation in areas of plaque development compared to control mice (67).

Even though the stenosis induced by atherosclerosis can restrict blood flow and thus induces CVD, the main mechanism implied in those diseases seems to be atherothrombosis. Indeed, following plaque rupture, prothrombotic materials (collagen and tissue factor) are exposed to the blood coagulation system leading to thrombus formation, decreased blood flow and CVD onset (39). Human postmortem studies showed that thrombi that form on disrupted plaques (e.g., asymptomatic coronary disease) appear small and non-occlusive (91). Animal models that can recapitulate spontaneous rupture of atherosclerotic lesions are very rare. To circumvent this issue, two experimental animal models have been developed to study platelet mechanism in thrombosis-induced plaque rupture. A model of ultrasound-induced plaque injury and a model of acute plaque rupture using a suture needle have been developed to test antiplatelet drugs in mice (92, 93). Ultrasound treatment resulted in a fissure at the shoulder region of the plaque leading to plaque material exposure (collagen) and unstable thrombus formation. Unlike the ultrasound model, the needle model is characterized by a frank rupture with larger and stable thrombi. At the site of plaque rupture, smaller thrombi were observed after P2Y12 or thrombin or integrin αIIbβ3 inhibition in both models (92–94). The role of GPVI seems to be more pronounced in the ultrasound model presumably due to a higher amount of thrombin generated in the needle model (93). The role of the different platelet molecules in mouse atheroprogression has been summarized in Table 1.

**Table 1:** A comprehensive analysis of platelet mechanisms in atheroprogression in mice (↓ decrease, ↑ increase, = no effect on plaque development).

**Molecule targeted**	**Animal model**	**Plaque development effect**	**References**
PGI_2_	ApoE^−/−^IP^−/−^ (chow diet)	↑	(81)
GPIbα	ApoE^−/−^+ Fab anti-GPIbα (HFD up to 18 weeks)	↓	(43)
	Ldlr^−/−^GPIbα^−/−^ (HFD for 16 weeks)	↓	(48)
	Ldlr^−/−^ hIL4R/GPIbα (HFD for 16 weeks)	**=**	(48)
vWF	Ldlr^−/−^vWF^−/−^ (HFD up to 22 weeks)	↓	(49)
αIIbβ3	ApoE^−/−^GPIIb^−/−^ (HFD for 8 at 12 weeks)	↓	(53)
GPVI	ApoE^−/−^+ Fab anti-GPVI (HFD for 12 weeks)	↓	(55)
PAR4	ApoE^−/−^PAR4^−/−^ (HFD for 5 or 10 weeks)	**=**	(74)
CD40L	ApoE^−/−^CD154^−/−^ (chow diet)	↓	(75)
CD40	ApoE^−/−^CD40^−/−^ (HFD for 4 weeks)	↓	(79)
PF4	ApoE^−/−^PF4^−/−^ (HFD for 10 weeks)	↓	(76)
RANTES	Ldlr^−/−^+ RANTES inhibitor (HFD up to 22 weeks)	↓	(77, 78)
TXA_2_	ApoE^−/−^+ S18886 or aspirin (chow diet)	↓	(80)
	ApoE^−/−^COX-1^−/−^ (HFD for 8 weeks)	↓	(82)
	Ldlr^−/−^+ aspirin (chow diet or HFD up to 26 weeks)	↓	(83–85)
	ApoE^−/−^+ aspirin (chow diet or HFD up to 12 weeks)	**=**	(70, 80)
	ApoE^−/−^+ aspirin long treatment (HFD up to 16 weeks)	↑	(87)
CalDAG-GEFI	CalDAG-GEFI^−/−^ in hematopoietic cells (HFD for 12 weeks)	↓	(67)
P2Y12	ApoE^−/−^P2Y12^−/−^ (HFD up to 20 weeks)	↓	(65, 67)
	ApoE^−/−^+ Clopidogrel (chow diet or HFD between 8 at 12 weeks)	↓	(68, 70)
	ApoE^−/−^+ Clopidogrel (HFD for 6 months)	**=**	(69)
	ApoE^−/−^+ Ticagrelor (HFD for 20 weeks)	↓	(71, 72)
	ApoE^−/−^+ Ticagrelor (HFD for 12 weeks)	**=**	(73)

The protective role of these drugs is difficult to assess in human clinical trials since atherosclerosis by itself is almost always asymptomatic. Thereby, their efficacy is studied in CVD with an atherosclerotic origin such as myocardial infarction (MI) and stroke.

## Platelets Contribute to Thrombo-Inflammation During Stroke

According to the World Health Organization, an estimated 7 million people died from stroke worldwide in 2016. Stroke represents the second most common cause of death and the third most common cause of disability (95). Strokes have mainly an ischemic origin (70%) and occur when an artery that supplies blood to the brain is blocked by a blood clot (96). Hemorrhagic stroke accounts for 15% of all strokes but they are responsible for about 40% of all stroke deaths (National Stroke Association). An ischemic stroke can occur in two ways: embolic stroke caused by thromboembolism of cardiac origin or thrombotic stroke with *in-situ* blood clot. Atherosclerosis in major intracranial arteries leads to changes ranging from minor wall thickening to luminal stenosis, and is one of the most common causes of stroke worldwide (97). The middle cerebral arteries are the most common lesion site, followed by the basilar artery, the internal carotid arteries, and the intracranial vertebral arteries (98). Intracranial atherosclerotic disease may occur concomitantly with systemic atherosclerosis.

The current treatments for acute ischemic stroke are the use of a thrombolytic agent as recombinant tissue plasminogen activator (t-PA) (99) and mechanical thrombectomy (100). Nevertheless, in some patients, the recanalization by thrombolysis is not efficient and the persisting thrombus leads to severe brain damage. Recent clinical findings show that clots retrieved from stroke patients have a thick compact outer shell enriched in NETs and fibrin which might contribute to reperfusion resistance (101, 102). Following thrombolysis, the overall recanalization rate is 46% (103). However, reocclusion after initial recanalization occurs in 14–34% of patients and is associated with clinical deterioration and poor outcome (104–106). Reocclusion has been attributed to increased platelet aggregation caused by the local thrombus and endothelial injury. Thus, the start of antiplatelet therapy early after thrombolysis might reduce the risk of reocclusion and thereby improve functional outcome.

Animal models were used to decipher platelets involvement in stroke. The most common stroke animal model used is the transient middle cerebral artery occlusion (tMCAO) in mice and rats. Thrombocytopenic mice were submitted to transient occlusion of the middle cerebral artery, and 24 h after ischemia/reperfusion, infarct area was determined (107). Platelet depletion did not significantly affect the lesion area, but thrombocytopenic mice presented multiple hemorrhagic foci in the lesion whereas none were observed in mice with normal platelet count. Nevertheless, platelet adhesion and activation have been investigated in several stroke studies in mice. Blockade or genetic deficiency of GPIbα improves stroke outcome without hemorrhagic transformation after tMCAO (108). Similarly, vWF deficiency is also associated with smaller infarct volumes and no bleeding was observed after tMCAO in those mice (109). Interestingly, the role of the GPIbα-vWF axis in hemostasis can be decoupled from the one in brain injury highlighting a proinflammatory role of GPIbα. The contribution of platelet activation receptors has been investigated during ischemia-reperfusion injury after tMCAO. Inhibition or genetic deficiency of GPVI, the collagen and fibrin receptor, has been shown to reduce the infarct volume and to improve stroke outcome (108, 110). Supporting the beneficial role of blocking the collagen receptor during a stroke, a recent study showed that GPVI inhibition plus intravenous infusion of rt-PA is safe in term of bleeding and has a better outcome than rt-PA alone (111). GPVI seems an attractive target in stroke since (i) it's only expressed on platelets, (ii) patients with a GPVI deficiency have no or mild bleeding phenotype (112), (iii) GPVI inhibition leads to a significant reduction of thrombus formation (113, 114), (iv) a novel humanized Fab anti GPVI (ACT017) in healthy donors didn't show bleeding complications (15). Overall, targeting GPVI in thrombosis can be a novel approach and compared to the current antiplatelet drugs, GPVI inhibition does not compromise hemostasis. Nevertheless, other larger studies need to be conducted in patients with CVD to validate the use of anti-GPVI antibodies. Notably, the protective effect of GPIbα and GPVI inhibition can also be observed in aged mice presenting comorbid factors such as atherosclerosis, diabetes or hypertension (115). Indeed, platelets from patients with comorbid factors are in a hyperactivated state (116). Enhanced platelet intracellular calcium responses to LDL cholesterol have been observed in diabetic patients with and without hypertension (117). Similarly, platelets from diabetic patients have been reported to have reduced sensitivity to prostacyclin (118) and hyperaggregate in response to platelet agonists (119). Other changes in platelets from diabetic patients include an increased expression of some platelet receptors GPIbα and αIIbβIIIa (120) and an alteration of platelet membrane fluidity (121). Therefore, studies including comorbid factors should be performed to further assess the validity of future antiplatelet drugs in the context of CVD.

Other platelet receptors are involved in platelet activation during the ischemia-reperfusion mouse model. The thrombin PAR4 receptor is expressed not only in platelets but also in the central nervous system (122). In a transient stroke mouse model, its systemic deletion reduces the brain infarct volume and attenuates cognitive function deficit (123). Intravital microscopy studies showed fewer platelet/EC interactions in PAR4^−/−^ mice compared to control mice. Thus, PAR4 deficiency seems to be neuroprotective in transient middle cerebral artery occlusion, partially through the attenuation of cerebral microvascular inflammation. In addition, mice deficient for Gα_i2_, G protein downstream of the ADP receptor P2Y12, were subjected to tMCAO, then functional outcome and infarct size were assessed 24 h later (124). Gα_i2_ deficiency leads to a reduced lesion area and better functional outcome than control mice. Apart from platelet adhesion and activation, platelet granule secretion contributes to stroke. Mice lacking platelet α-granules (Nbeal2^−/−^) and mice lacking platelet dense granules (Unc13d^−/−^) showed a higher mortality rate due to intracranial hemorrhage. Nevertheless, the surviving animals developed significantly smaller brain infarctions and had a better outcome compared to WT mice (125). Platelet aggregation mediated by the integrin αIIbβ3 seems dispensable at sites of ischemia/reperfusion injury. Blockade of αIIbβ3 integrin increases intracranial hemorrhage risk in tMCAO mice (108, 126) and among the surviving mice, the treatment did not show any improvement (115) suggesting that platelet aggregation is dispensable for brain injury but still important for securing hemostasis in tMCAO.

**Figure 1:**
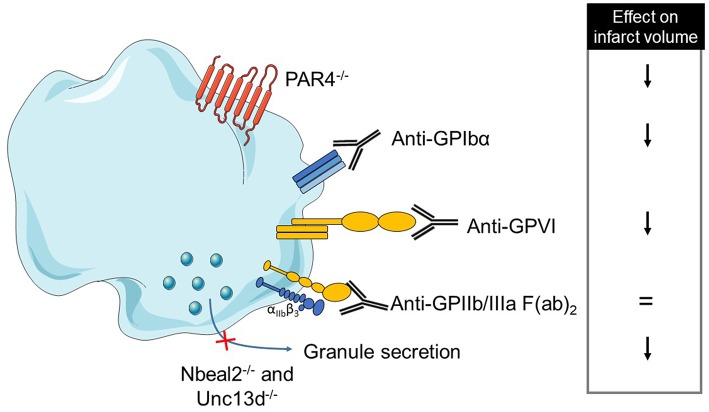
Overview of platelet receptors inhibition involved in stroke. Pharmalogical or genetic inhibition of platelet receptors or secretion shows a decrease of brain infarct (↓ decrease, = no effect).

Overall, these data suggest a deleterious role of platelet adhesion, activation and secretion in the stroke pathophysiology. On the opposite, platelet aggregation, which plays a crucial role in thrombus formation, is not required for stroke progression. Thus, the non-classical role of platelets, through their pro-inflammatory properties, may prevail in stroke disease. A comprehensive table summarizes platelet mechanisms studied in tMCAO models (Table 2A and Figure 1).

**Table 2:** **(A)** A comprehensive analysis of platelet mechanisms in tMCAO mouse model and **(B)** in stroke patients (↓ decrease, ↑ increase, = no effect on infarct area or bleeding risk).

**A**			
**Molecule targeted**	**Animal model**	**Effect on infarct area**	**References**
Platelets	Thombocytopenia	**=**	(107)
α granules	Nbeal2^−/−^	↓	(125)
Dense granules	Unc13d^−/−^	↓	(125)
PAR4	PAR4^−/−^	↓	(123)
Gα_i2_	Gαi2^fl/fl^/PF4-Cre	↓	(124)
GPIbα	Antibody anti-GPIbα	↓	(108, 126)
GPVI	Antibody anti-GPVI	↓	(108)
αIIbβ3	Antibody anti-GPIIb/IIIa	↓	(108)

**B**				
**Molecule targeted**	**Clinical trial**	**Effect**	**Bleeding**	**References**
TXA_2_	JPPP Aspirin	↓	↑	(127)
	ARRIVE Aspirin	**=**	↑	(128)
	ASPREE Aspirin	**=**	↑	(129)
	ARTIS Early aspirin addition to thrombolysis	**=**	↑	(130, 131)
P2Y12	CAPRIE Clopidogrel vs. aspirin - Ischemic stroke patients	**=**	↑	(132)
	CHARISMA Clopidogrel + aspirin vs. aspirin alone	**=**	↑	(133)
	MATCH Clopidogrel + aspirin vs. aspirin alone	**=**	↑	(134)
	CHANCE Clopidogrel + aspirin vs. aspirin alone	↓	**=**	(135, 136)
	COMPRESS Clopidogrel + aspirin vs. aspirin alone	**=**	↑ Tendency	(137)
	POINT Clopidogrel + aspirin vs. aspirin alone	↓	↑	(138, 139)
	SOCRATES Ticagrelor vs. aspirin	**=**	**=**	(140, 141)
	PRINCE Ticagrelor vs. clopidogrel (+ aspirin)	↓ Tendency	?	(142, 143)
	TARDIS Aspirin, clopidogrel and dipyridamole vs. guideline treatment	**=**	↑	(144)
αIIbβ3	abESTT-II Abciximab	**=**	↑	(145)
	SaTIS Tirofiban vs. placebo	**=**	**=**	(146)

In humans, several antiplatelet drugs have been tested in stroke outcome. Among them, numerous studies have investigated the benefits and risks of aspirin for primary prevention in population at risk, during the acute management of cardiovascular events and in secondary prevention among patients with CVD. In elderly populations, the risk of CVD is higher suggesting an increased benefit of aspirin administration in primary prevention for cardiovascular events. However, increased bleeding risk has also been observed in this population (147). According to a meta-analysis of aspirin primary prevention studies, reported by the Antithrombotic Trialists' Collaboration, aspirin did not reduce the risk of stroke. In this meta-analysis, aspirin non-significantly reduced the risk of ischemic stroke but increased non-significantly the risk of hemorrhagic stroke (148). The JPPP (Japanese Primary Prevention Project) clinical trial was designed to assess whether primary prevention would reduce the risk of non-fatal stroke in elderly Japanese patients (127). It appears that aspirin seems to reduce the non-fatal ischemic stroke risk, but it tends to increase the risk of hemorrhagic stroke. Recently, the ARRIVE (Aspirin to Reduce Risk of Initial Vascular Events) clinical trial investigated the benefits and risks of enteric-coated aspirin used in primary prevention of cardiovascular events, in patients with an average cardiovascular risk (128). Aspirin did not lower the risk of major cardiovascular events nor stroke incidence. Moreover, rates of gastrointestinal bleeding events and some other minor bleeding events were higher in the aspirin treatment group with no difference in the incidence of fatal events. The results of the ASPREE (Aspirin in Reducing Events in the Elderly) clinical trial investigating the role of aspirin in primary prevention of CVD were published recently (129). They confirm that the use of low-dose aspirin as a primary prevention strategy in older adults results in a significantly higher risk of major hemorrhage and did not trigger a significantly lower risk of CVD than placebo.

Low-dose aspirin efficacy has been widely established in secondary prevention trials, in which the benefits of reducing ischemic stroke rates have outweighed the risk of hemorrhage (149, 150). A meta-analysis including eight clinical trials involving more than 40,000 participants concluded that antiplatelet therapy with aspirin started within 48 h of the onset of ischemic stroke reduced the risk of early recurrent ischemic stroke without a major risk of early hemorrhagic complications (151). Moreover, long-term outcomes were also improved. The ARTIS (Antiplatelet therapy in combination with rt-PA Thrombolysis in Ischemic Stroke) clinical trial compared the effects of early intravenous aspirin addition to thrombolysis with standard treatment without aspirin (130). Patients with acute ischemic stroke treated with rt-PA thrombolysis were randomly assigned to intravenous aspirin within 90 min after the start of thrombolysis treatment or to no additional treatment. In addition, in both groups, oral antiplatelet therapy was started 24 h after thrombolysis treatment. This trial concluded that early administration of intravenous aspirin does not improve outcome at 3 months and increases the risk of intracranial hemorrhage without evidence of a beneficial effect on early neurological deterioration (131).

Current guidelines for the early management of patients with acute ischemic stroke, from the American Heart Association and the American Stroke Association, recommend starting aspirin administration 24 h after thrombolysis (152). However, the overall effect of aspirin in acute ischemic stroke is weak and better acute therapies are therefore necessary.

Over the years, several other antiplatelet agents were developed and then tested in clinical trials. A comprehensive table summarizes the different clinical trials that assessed the efficacy of antiplatelet agents in stroke outcome (Table 2B).

## Platelets Contribute to Myocardial Injury

Acute coronary syndrome (ACS) occurs when the blood flow is decreased or stopped in coronary arteries, leading to tissue damage ranging from ischemia to infarction. This defect of blood supply is mainly due to atherosclerotic plaque growth and rupture, followed by a thrombus formation in coronary artery (153). ACS is commonly divided into myocardial infarction (MI) and unstable angina, considered to be an imminent precursor of MI. Biomarkers of cardiac tissue damage such as troponin and creatine-kinase myocardial band (CK-MB) are used to classify ACS into unstable angina or MI, the latter one presenting such blood biomarkers. A MI can have several consequences such as heart failure, an irregular heartbeat or a cardiac arrest. According to the World Health Organization, in 2012 an estimated 7.5 million people died from MI worldwide. Survivors of MI are at increased risk of recurrent infarctions and have an annual death rate of 5%, representing a 6-time increase compared to people without coronary heart disease. MI can also be classified into ST-segment elevation myocardial infarction (STEMI) and non-STEMI (NSTEMI) according to the patient electrocardiogram. STEMI caused by a complete coronary vessel occlusion, and NSTEMI due to a partial artery occlusion, represent respectively 30 and 70% of all MI (154). Since unstable angina has similar pathophysiology to NSTEMI, they are referred together as non-ST-segment elevation ACS (NSTE-ACS) and are grouped for care management decisions. Current guidelines recommend an immediate treatment of ACS, but due to different pathophysiology between STEMI and NSTE-ACS, separate guidelines were edited. Guidelines for the management of NSTE-ACS recommend a pharmacological treatment of ischemia (via decreasing myocardial oxygen demand or increasing myocardial oxygen supply) (155). Guidelines for the treatment of STEMI recommend an immediate recanalization of coronary arteries via reperfusion therapies such as primary percutaneous coronary intervention and/or fibrinolysis strategy (156). Even though recanalization is necessary to provide oxygen and nutrients to the ischemic area, reperfusion by itself also exacerbates myocardial damage (157). This pathologic process is named ischemia-reperfusion (IR) injury. For long-term therapies, both guidelines agree to strongly recommend the use of antiplatelet agents.

Indeed, the atherosclerotic origin of MI led to numerous studies deciphering the platelet involvement in MI. A lower platelet count or no significant difference of the platelet count was observed between MI patients and stable angina or healthy donors (158, 159). However, the mean platelet volume reflecting platelet activation was higher in MI patients compared to stable coronary artery disease patients at the time of acute event (158). Increased levels of P-Selectin and CD63-exposing platelet microparticles have been found in MI patients (160). Plasma levels of vWF and serotonin are increased in patients with coronary artery syndrome (161, 162) highlighting the role of platelet activation in myocardial injury. Moreover, platelet-leucocyte aggregates are an early marker of acute MI and are also associated with myocardial no-reflow in STEMI patients (163, 164). Ventricular wall rupture is a fatal complication of acute MI and platelets seem to be involved in this phenomenon since an intramural thrombus was observed within the infarcted myocardium (165). Platelets potential involvement in this process was confirmed by their depletion which reduced the rate of myocardial wall rupture from 46 to 0% (166). Several studies investigated platelet mechanisms involved in MI and in myocardial (IR) injury.

Platelet activation is commonly observed in numerous pathologies, including MI (158). This phenomenon is mediated by specific platelet receptors that are involved in adhesion and activation. Inhibition of GPIbα–involved in platelet tethering—via a Fab anti-GPIbα did not change the infarct volume per the area at risk (INF/AAR) (167). In accordance with the results of GPIbα inhibition, depletion of the phospholipase D1 (PLD1), enzyme involved in GPIbα dependent αIIbβ3 activation, did not protect mice from myocardial IR injury (167). These findings suggest that GPIbα platelet receptor is dispensable in MI pathophysiology. On the contrary, mice deficient for the Fc receptor γ chain (FcRγ) coupled to GPVI were protected from myocardial IR injury with smaller infarct size and reduced leucocyte recruitment in the injured area (168). This was confirmed by pharmacological inhibition of the collagen receptor GPVI via a Fab anti-GPVI or soluble GPVI-Fc (Revacept) in a mouse model of the left anterior descending artery ligation with reperfusion. Inhibition of GPVI led to a reduced infarct size (167, 169). These findings suggest that a therapeutic strategy targeting GPVI could be a valuable approach in MI. This could be relevant in humans since it was recently described that patients with STEMI have an alteration of GPVI platelet signaling (170). Indeed, platelets from STEMI patients have an increased aggregation response compared to stable coronary artery disease patients. This could be due to the increased number of GPVI receptors observed in ACS patients (171). Therefore, studies focused on GPVI inhibition seem promising. Currently, a humanized Fab targeting GPVI without increasing the bleeding risk in healthy controls is developed and characterized (15) but their findings warrant further investigations in CVD patients under current antiplatelet drugs.

The contribution of other receptors in MI was studied such as CLEC-2, PAR receptors or P2Y12 in mice. Pharmacological inhibition of CLEC-2 via Fab administration did not decrease the INF/AAR in mice (167). The thrombin receptors, PAR1 and PAR4 receptors are expressed at the surface of platelets, but also by cardiomyocytes (172, 173). It has been showed that PAR1 deficiency did not affect the infarct size after myocardial IR injury (174). However, interestingly, PAR1^−/−^ mice had reduced cardiac remodeling and decreased impairment of left ventricle function compared to control mice. In contrast to previous findings, PAR1 antagonist (SCH 79797) administration was shown to reduce infarct size after myocardial IR injury in rats (175). This discrepancy could be explained by off-target effects of SCH 79797 or a PAR4 compensation. Genetic depletion of PAR4 led to the development of larger infarcts and more myocardial apoptosis compared to control mice (176). However, another study attributes a cardioprotective effect of PAR4 deletion after myocardial IR injury as INF/AAR was decreased in PAR4^−/−^ mice compared to control mice (177). The administration of PAR4 antagonists confirmed the previous findings. Indeed, PAR4 inhibition in rats decreased infarct size after myocardial IR injury (178). The P2Y12 receptor, which signaling is mediated by the G protein Gα_i2_, is involved in platelet activation. ${\text{G}\alpha }_{\text{i}2}^{-/-}$ deficient mice have a reduced INF/AAR ratio suggesting a protective effect of platelet activation inhibition (124). However, this G protein may interact not only with P2Y12, but also with additional G protein coupled receptor present in platelets. The use of clopidogrel, a P2Y12 antagonist, decreased platelet accumulation in ischemic myocardium and reduced the rupture rate from 45% in control to 10% in clopidogrel treated animals (165, 166). In a rat model of isolated hearts, the perfusion of platelets from acute MI patients enlarges infarct area (179) while the concomitant administration of cangrelor or abciximab decreases the infarct size. As opposed to clopidogrel, aspirin administration did not reduce infarct size nor the rupture rate in mice (165, 180) suggesting a relative hierarchy in the platelet receptors during MI.

Platelet degranulation, a marker of platelet activation, triggers the inflammatory responses by P-selectin exposure involved in leukocyte recruitment. Nbeal2^−/−^ and Unc13d^−/−^ mice lacking, respectively, alpha and dense granules did not show any alterations in infarct sizes, arguing against a significant role of degranulation in the pathophysiology of myocardial IR injury (167). Nevertheless, blockade or genetic deficiency of P-selectin can lead to smaller infarct sizes after myocardial infarction (181–183) suggesting a possible contribution of the endothelial P-selectin. Critical to platelet activation is calcium mobilization. Cytosolic Ca^2+^ concentration is regulated by two major proteins: STIM1 (endoplasmic reticulum Ca^2+^ sensor) and Orai-1 (Ca^2+^ channel). Genetic depletion in hematopoietic cells of STIM1 or Orai-1 did also not reduce the INF/AAR ratio (167) hinting at alternative platelet activation pathways.

**Figure 2:**
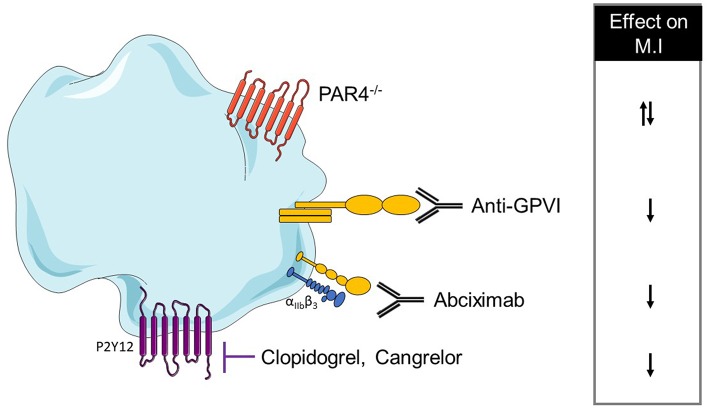
Overview of platelet receptors inhibition involved in myocardial infarction. Pharmalogical or genetic inhibition of platelet receptors shows a decrease of myocardial injury (↓ decrease, ↑ increase).

The final step of platelet activation is their aggregation and is mainly driven by αIIbβ3 activation. The inhibition of this integrin via Fab anti- αIIbβ3 administration did not alter the INF/AAR ratio when compared with control mice (167). In another study, αIIbβ3 inhibition by abciximab seems to reduce myocardial injury in isolated rat hearts through a reduction of platelet adhesion to the endothelium or leukocytes (179). This discrepancy can be probably explained by the different experimental models which can have a different impact on platelet activation. Platelet mechanisms in the MI model have been summarized in Table 3A and Figure 2.

**Table 3:** **(A)** A comprehensive analysis of platelet mechanisms in myocardial infarction (MI) mouse model and **(B)** in MI patients (↓ decrease, ↑ increase, = no effect on MI or bleeding risk).

**A**			
**Molecule targeted**	**Animal model**	**Effect on M.I**.	**References**
Platelets	Thrombocytopenia	↓	(166)
GPIbα	Fab anti-GPIbα	**=**	(167)
Pld1	Pld1^−/−^	**=**	(167)
GPVI	Antibody anti-GPVI	↓	(167, 169)
	Soluble GPVI-Fc	↓	(169)
FcRγ	FcRγ^−/−^	↓	(168)
CLEC-2	Fab anti-CLEC-2	**=**	(167)
PAR4	PAR4^−/−^	↑	(176)
	PAR4^−/−^	↓	(177)
	PAR4 antagonist	↓	(178)
Gα_i2_	G$\alpha_{\text{i}2}^{-/-}$	↓	(124)
αIIbβ3	Fab anti-GPIIb/IIIa Isolated hearts and perfusion of platelets from acute MI patients + Abciximab	**=** ↓	(167) (179)
δ granules	Nbeal2^−/−^	**=**	(167)
Dense granules	Unc13d^−/−^	**=**	(167)
Stim1	Stim1^−/−^	**=**	(167)
Orai1	Orai1^−/−^	**=**	(167)
TXA_2_	Aspirin	**=**	(165, 180)
P2Y12	Clopidogrel	↓	(165, 166)
	Isolated hearts and perfusion of platelets from acute MI patients + Cangrelor		
		↓	(179)

**B**				
**Molecule targeted**	**Clinical trial**	**Effect**	**Bleeding**	**References**
P2Y12	CURE Clopidogrel + aspirin vs. aspirin alone	↓	↑	(184)
	TRITON-TIMI-38 Prasugrel vs. clopidogrel (+ aspirin)	↓ Prasugrel	↑ Prasugrel	(185)
	PLATO Ticagrelor vs. clopidogrel (+ aspirin)	↓ Ticagrelor	**=**	(186)
	PEGASUS-TIMI-54 Ticagrelor + Aspirin vs. Aspirin alone	↓	↑	(187–189)
PAR1	The TRA 2P–TIMI 50 Vorapaxar vs. placebo (+ concomitant medical therapy)	↓	↑	(190)
	TRACER Vorapaxar vs. placebo (+ standard therapy)	↓	↑	(191)

In humans, several clinical trials tested platelet inhibitors in the context of MI. Currently, guidelines for the management of NSTE-ACS and STEMI patients recommend aspirin intake for long-term treatment for all patients without contraindications (148, 155, 156). Moreover, the dual antiplatelet treatment (DAPT) composed of aspirin plus a P2Y12 inhibitor is recommended. Indeed, the CURE (Clopidogrel in Unstable Angina to Prevent Recurrent Events) trial was designed to assess the efficacy of the combination of aspirin and clopidogrel compared to aspirin alone (184). Patients with NSTE-ACS were enrolled within 24 h after symptom onset, and either treated with the combination treatment or aspirin alone. The DAPT significantly reduced the composite rate of death from cardiovascular causes, non-fatal MI and stroke. The rate of each component of this composite outcome also tended to be lower in the DAPT group. However, the risk of major bleeding is increased among patients treated with clopidogrel. TRITON-TIMI-38 (Trial to Assess Improvement in Therapeutic Outcomes by Optimizing Platelet Inhibition with Prasugrel—Thrombolysis in Myocardial Infarction-38) is a phase III trial which enrolled patients with ACS (NSTE-ACS and STEMI) within 72 h after symptom onset (185). This trial was designed to compare the efficacy of prasugrel with clopidogrel, both associated with aspirin. The percentage of non-fatal MI (Clopidogrel: 9.5%; Prasugrel: 7.3%) and stent thrombosis (Clopidogrel: 2.4%; Prasugrel: 1.1%) was significantly reduced in patients treated with prasugrel, but it caused more life-threatening bleedings than clopidogrel treatment (from 0.9 to 1.4%). In conclusion, this trial showed that prasugrel is more effective at preventing ischemic events than clopidogrel in patients with NSTE-ACS or STEMI. However, this beneficial effect is accompanied by an increased rate of major bleeding. The PLATO (Study of Platelet Inhibition and Patient Outcomes) clinical trial aimed to determine whether, combined to aspirin, ticagrelor is more efficient than clopidogrel in patients with ACS, enrolled within 24 h after symptom onset (186). Patients receiving ticagrelor had a significantly lower MI event rate (5.8%) compared to clopidogrel-treated patients (6.9%). The rate of death from any cause is also significantly lower for patients treated with ticagrelor (ticagrelor: 4.5%; clopidogrel: 5.9%). It is important to note that no difference in life-threatening bleeding was observed between the two treatment groups (ticagrelor: 5.8%; clopidogrel: 5.8%). This trial found that, in patients who have an NSTE-ACS or STEMI, treatment with ticagrelor compared to clopidogrel significantly reduced the rate of death from vascular causes, myocardial infarction, or stroke without an increase in the rate of overall major bleeding.

These results were later confirmed by the PEGASUS-TIMI-54 (Prevention of Cardiovascular Events in Patients With Prior Heart Attack Using Ticagrelor Compared to Placebo on a Background of Aspirin–Thrombolysis In Myocardial Infarction 54) trial which enrolled patient who had a MI 1 to 3 years earlier (187). Patients either received ticagrelor plus aspirin or aspirin alone. The ticagrelor treatment significantly reduced MI event rate (from 5.25 to 4.53%) (188). Moreover, this protective effect is consistent over time and this trial supports the use of prolonged therapy in patients who continue to tolerate this antiplatelet agent (189). Current guidelines recommend delivering DAPT to NSTE-ACS and STEMI patients, with aspirin plus ticagrelor or prasugrel (155). Clopidogrel can be administered to ACS patients who cannot receive the two previous antiplatelet agents.

Vorapaxar, a PAR1 inhibitor, has been tested in clinical trials. The TRA 2P–TIMI 50 (Thrombin Receptor Antagonist in Secondary Prevention of Atherothrombotic Ischemic Events—Thrombolysis in Myocardial Infarction 50) clinical trial enrolled patients who had a history of atherosclerosis within the previous 2 weeks to 12 months (190). Patients were randomly assigned to either vorapaxar treatment or placebo. All concomitant medical therapy, including the use of other antiplatelet agents, was managed by the clinicians who were responsible for the care of the patients. Patients receiving vorapaxar had a reduced rate of MI event (from 6.1 to 5.2%) but presented an increase in major bleeding (from 11.1 to 15.8%). This trial assessed that inhibition of PAR-1 with vorapaxar reduced the risk of cardiovascular death or ischemic events in patients with stable atherosclerosis. However, it increased the risk of moderate or severe bleeding, including intracranial hemorrhage. The findings of TRA 2P–TIMI 50 clinical trial were confirmed by the TRACER (Thrombin Receptor Antagonist for Clinical Event Reduction in Acute Coronary Syndrome) trial. Investigators aimed to compare vorapaxar administration with placebo, in addition to standard therapy, in patients suffering from NSTE-ACS (191). The main result observed is a decreased rate of MI event for patients treated with vorapaxar. These data support the use of vorapaxar in MI secondary prevention since it provided net clinical benefit in patients at low risk for bleeding but high risk for ischemic events, as it especially prevented stent thrombosis after MI. However, the safety and efficacy of vorapaxar in STEMI patients have not been investigated yet. These clinical findings have been summarized in Table 3B.

## Platelets Participate to the Development of the Abdominal Aortic Aneurysm (AAA)

Abdominal aortic aneurysm (AAA) is a permanent and irreversible localized dilatation of the infrarenal segment of the abdominal aorta caused by the degradation and remodeling of the layers of the vessel wall and a chronic wall inflammation. AAA can extend along the aorta (fusiform), or be localized (sacciform). Major AAA risk factors are age, atherosclerosis, hypertension, male gender, and smoking. In western countries, AAA incidence is ~0.4–0.67% annually and reaches 5–10% of men and 1% of women over 65 years old (192). This pathology is mainly asymptomatic and aneurysm rupture leads to death. Endovascular and open repair of AAA remains the only effective treatments. Nevertheless, many pharmacological therapies are still under investigation like statins, angiotensin receptor blockers and anti-platelets drugs (193).

AAA is characterized by chronic inflammation with a large degradation of elastin and collagen fibers. It results in the proteolytic activity of matrix-degrading proteinases including matrix metalloproteinases (MMPs) leading to aorta dilatation. Reduced vascular wall thickness and the lack of tissue repair are associated with vascular smooth muscle cells apoptosis. The adventitia neovascularization induces inflammatory cells (lymphocytes, neutrophils and macrophages) infiltration in the aortic vessel wall, maintaining a continuous level of inflammation. This process contributes to the intra-luminal thrombus (ILT) formation (194), which involves platelets and coagulation activation. Overall, the ILT thromboinflammatory status contributes to the outward remodeling and eventually to the disruption of wall integrity (195, 196).

The ILT is structured in multilayers. In AAA patients, ILT is often organized in three layers—luminal (in contact with the blood), medial and abluminal (in contact with the wall). Luminal ILT layer is biologically active and enriched in platelets, neutrophils, red blood cells and a dense fibrin network (197). The ILT has also been shown to contain weak pathogens like *Porphyromonas gingivalis* (Pg) which contribute to leukocyte recruitment (198). On the opposite side, the abluminal layer has a marked fibrinolytic activity and contains few cells (199, 200). ILT evolution can lead to vessel wall weakness due to the high concentrations of reactive oxygen species (ROS), proteases and cytokines. Indeed, a study showed that ILT thickness is correlated with AAA diameter and MMP9 expression (201). The main specificity of the ILT in AAA is its non-healing property. The continuous release of neutrophil-derived proteases from these thrombi prevents vascular healing. Indeed, the re-endothelialization and adherence of mesenchymal stem cells are prevented by neutrophil proteases (202). This protease-rich thrombus is considered as the driving force in vessel wall rupture leading to death (203). However, ILT formation mechanisms in AAA are so far unknown.

Observational studies based on human tissue samples from AAA patients provide information at the late stage of the disease. In order to understand the mechanisms in the early steps, different animal models have been used, including mice and rats. The role of platelets and coagulation in ILT formation during AAA has been recently reviewed elsewhere (194). To study the role of platelets in AAA, two major models were used in mice and rats. The hypertension model, induced by angiotensin II in ApoE^−/−^ or Ldlr^−/−^ mice, reproduces important features of human AAA with inflammation, smooth muscle cells apoptosis and macrophage infiltration. However, aneurysms formed have a suprarenal location and abluminal thrombus formation occurs after an aortic dissection due to a false channel (204).

The main model in rats consists of elastin degradation via elastase perfusion and presents the same characteristics as the first model. However, in this model, aneurysms have an infrarenal location and do not present a thrombus and hypertension (205). This model was also developed in mice (206). A recent study showed that administration of beta 3-aminopropionitrile fumarate salt (BAPN, inhibitor of lysyl oxidase) in the drinking water of elastase-treated mice resulted in ILT formation (207). Both of these models (elastase and angiotensin II) do not recapitulate all human characteristics but they contribute to better understand the disease.

A xenograft rat model which consists of grafting decellularized aorta of guinea pig into rat aorta has been shown to present an ILT (208). With this model, abciximab treatment (platelet aggregation inhibitor) reduces the aneurysmal diameter and ILT activities accompanied by fewer P-selectin expression and reduced vessel wall degradation. These results suggest that platelets are involved in the thrombus biological activity and aneurysm development (197). Similar results were observed in rats after 10 and 42 days of AZD6140 treatment, a P2Y12 receptor antagonist. A reduced ILT was observed as well as decreased MMP-9, MMP-2 expression, leukocyte infiltration, media and elastin preservation (209).

**Figure 3:**
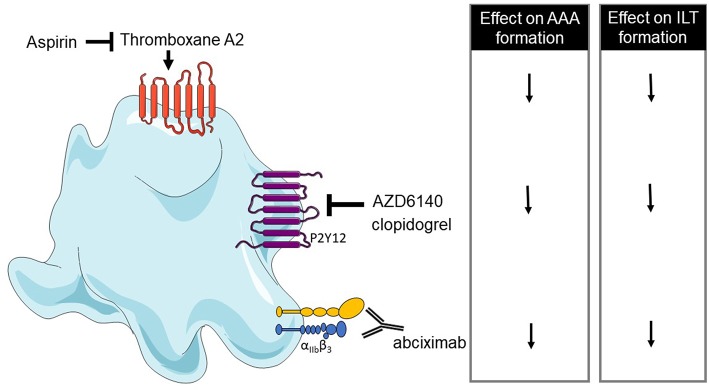
Overview of platelet receptors inhibition involved in abdominal aortic aneurysm (AAA). Pharmalogical inhibition of platelet receptors shows a decrease of AAA formation and intraluminal thrombus (ILT) formation (↓ decrease).

Other studies with angiotensin II mouse model have shown that clopidogrel treatment (inhibitor of P2Y12), or aspirin (inhibitor of COX-2) reduces the macrophage infiltration, MMP2 and ROS production, suggesting that platelets play a role in vascular inflammation during AAA progression (210, 211). A reduction of thrombi, uPA, t-PA, and PF4 in the aorta was also observed in aspirin or clopidogrel-treated mice but these treatments have no effect on aorta diameter on established AngII-aneurysm model (211). However, clopidogrel administration in the early steps of AAA decreases the aorta diameter (210). The same treatments in patients emphasize that anti-platelet treatments can reduce AAA progression and rupture or dissection (211). Low-dose of aspirin can prevent the progression of AAA measuring from 40 to 49 mm and no decrease of AAA growth was observed in AAAs measuring <40 mm (212). The use of a P2Y12 receptor inhibitor as ticagrelor treatment revealed a lack of difference in AAA size compared to placebo-treated subjects, suggesting that ticagrelor has no effect on the development of small AAAs. However, in this study, most of the patients did not present an ILT (213). These results have been summarized in Table 4 and Figure 3.

**Table 4:** **(A)** A comprehensive analysis of platelet mechanisms in abdominal aortic aneurysm (AAA) animal models and **(B)** in AAA patients (↓ decrease, ↑ increase, = no effect on intraluminal thrombus ILT or aneurysm diameter).

**A**			
**Molecule targeted**	**Animal model**	**Effect on ILT**	**References**
αIIbβ3	Xenograft rats model + abciximab	↓	(197)
P2Y12	Xenograft model + AZD6140	↓	(209)
	Angiotensin II + Clopidogrel	↓	(210)
P2Y12 and TXA_2_	Angiotensin II + Clopidogrel and Aspirin	↓	(211)

**B**			
**Molecule targeted**	**Clinical trial**	**Effect on aneurysm diameter**	**References**
TXA_2_	Aspirin	↓ (between 40 and 49 mm)	(212)
P2Y12	Ticagrelor	**=**	(213)

Nowadays, there is no definite treatment to decelerate or stop AAA progression. Nevertheless, clinical and animal studies mentioned above provide additional information on the effect of platelets in AAA development, opening up treatment prospects that may be in the long term substitutes of heavy and invasive surgery.

## Conclusions and Perspectives

Over the past years, the field of platelets gained a lot of attention in their contribution to vascular diseases. Platelet biology is at the crossroads of several clinical specialties (cardiology, neurology, pulmonology). The current use of antithrombotic drugs, aspirin, and P2Y12 antagonists, is based on their inhibitory effect on platelet aggregation. While those drugs show a beneficial effect in CVD, they are still associated with some bleeding risks. Recent studies provided a new understanding of the role of platelets in vascular inflammation that extends beyond their role in aggregation. The development of future anti-platelet drugs will need to take into account the role of platelets in inflammation. In addition, as most of the thrombosis and inflammatory studies are conducted in healthy vessels, it is important to keep in mind that the identified mechanisms need to be validated in models relevant to CVD. Importantly, there is significant inter-individual variability of antiplatelet therapy responses among patients with CVD highlighting the need for tailored therapies to each individual. Central to this approach is the development of robust assays that can determine platelet reactivity in a patient-specific manner.

## Author Contributions

HL did the literature research and wrote the first draft of the review. KY wrote a part of the manuscript. RM designed the tables. YB contributed to the idea of the manuscript, wrote parts of the manuscript, and provided critical feedback. All the authors reviewed the manuscript and approved the submitted version.

### Conflict of Interest Statement

The authors declare that the research was conducted in the absence of any commercial or financial relationships that could be construed as a potential conflict of interest.

## Abbreviations

AAA: abdominal aortic aneurysm
ACS: acute coronary syndrome
BAPN: beta 3-aminopropionitrile fumaralt salt
CalDAG-GEFI: calcium and diacylglycerol-regulated guanine nucleotide exchange factor I
CLEC2: C-type lectin 2
COX-1: cyclooxygenase-1
CVD: cardiovascular diseases
DAG: diacylglycerol
DAPT: dual antiplatelet treatment
EC: endothelial cells
ECM: extracellular matrix
FcRγ: Fc receptor γ chain
GPCRs: G protein-coupled receptors
GPIbα: glycoprotein Ibα
GPVI: glycoprotein VI
ILT: intra-luminal thrombus
IR: ischemia-reperfusion
ITAM: immunoreceptor tyrosine-based activation motif receptors
MI: myocardial infarction
MMP: matrix-degrading proteinases
NETs: neutrophil extracellular traps
oxLDL: oxidized low density lipoproteins
PAF: platelet-activating factor
PAR: protease-activated receptor
PDGF: platelet derived growth factor
Pg: *Porphyromonas gingivalis*
PKC: protein kinase C
PLA: platelet-leucocyte aggregates
PLD1: phospholipase D1
rt-PA: recombinant tissue plasminogen activator
ROS: reactive oxygen species
STEMI: ST-segment elevation myocardial infarction
tMCAO: transient middle cerebral artery occlusion model
TP: TXA2 receptor
TxA2: thromboxane A2
vWF: von Willebrand factor.

## References

[1] Osler W An account of certain organisms occurring in the liquor sanguinis. Monthly Microscopical J. (1874) 12:141–8. 10.1111/j.1365-2818.1874.tb01798.x

[2] Bizzozero J Ueber einen neuen formbestandtheil des blutes und dessen rolle bei der thrombose und der blutgerinnung. Arch Pathol Anat. (1882) 90:261–332. 10.1007/BF01931360

[3] Brill A Fuchs TA Chauhan AK Yang JJ De Meyer SF Köllnberger M. von Willebrand factor-mediated platelet adhesion is critical for deep vein thrombosis in mouse models. Blood. (2011) 117:1400–7. 10.1182/blood-2010-05-287623 20959603 PMC3056477

[4] von Brühl ML Stark K Steinhart A Chandraratne S Konrad I Lorenz M Monocytes, neutrophils, and platelets cooperate to initiate and propagate venous thrombosis in mice *in vivo*. J Exp Med. (2012) 209:819–35. 10.1084/jem.20112322 22451716 PMC3328366

[5] van der Meijden PEJ Heemskerk JWM. Platelet biology and functions: new concepts and clinical perspectives. Nat Rev Cardiol. (2019) 16:166–79. 10.1038/s41569-018-0110-0 30429532

[6] Harper MT Poole AW. Diverse functions of protein kinase C isoforms in platelet activation and thrombus formation. J Thromb Haemost. (2010) 8:454–62. 10.1111/j.1538-7836.2009.03722.x 20002545

[7] Jin J Daniel JL Kunapuli SP Molecular basis for ADP-induced platelet activation. II. The P2Y1 receptor mediates ADP-induced intracellular calcium mobilization and shape change in platelets. J Biol Chem. (1998) 273:2030–4. 10.1074/jbc.273.4.2030 9442040

[8] Hollopeter G Jantzen HM Vincent D Li G England L Ramakrishnan V. Identification of the platelet ADP receptor targeted by antithrombotic drugs. Nature. (2001) 409:202–7. 10.1038/35051599 11196645

[9] Nieswandt B Bergmeier W Eckly A Schulte V Ohlmann P Cazenave JP. Evidence for cross-talk between glycoprotein VI and Gi-coupled receptors during collagen-induced platelet aggregation. Blood. (2001) 97:3829–35. 10.1182/blood.V97.12.3829 11389023

[10] Violi F Basili S Berger JS Hiatt WR Antiplatelet therapy in peripheral artery disease. Handb Exp Pharmacol. (2012) 210:547–63. 10.1007/978-3-642-29423-5_22 22918746

[11] van der Meijden PE Munnix IC Auger JM Govers-Riemslag JW Cosemans JM Kuijpers MJ. Dual role of collagen in factor XII-dependent thrombus formation. Blood. (2009) 114:881–90. 10.1182/blood-2008-07-171066 19372258

[12] Kahn ML Nakanishi-Matsui M Shapiro MJ Ishihara H Coughlin SR. Protease-activated receptors 1 and 4 mediate activation of human platelets by thrombin. J Clin Invest. (1999) 103:879–87. 10.1172/JCI6042 10079109 PMC408153

[13] Inoue O Suzuki-Inoue K McCarty OJ Moroi M Ruggeri ZM Kunicki TJ. Laminin stimulates spreading of platelets through integrin alpha6beta1-dependent activation of GPVI. Blood. (2006) 107:1405–12. 10.1182/blood-2005-06-2406 16219796 PMC1895394

[14] Schaff M Tang C Maurer E Bourdon C Receveur N Eckly A. Integrin α6β1 is the main receptor for vascular laminins and plays a role in platelet adhesion, activation, and arterial thrombosis. Circulation. (2013) 128:541–52. 10.1161/CIRCULATIONAHA.112.000799 23797810

[15] Voors-Pette C Lebozec K Dogterom P Jullien L Billiald P Ferlan P. Safety and tolerability, pharmacokinetics, and pharmacodynamics of ACT017, an antiplatelet GPVI (Glycoprotein VI) Fab. Arterioscler Thromb Vasc Biol. (2019) 39:956–64. 10.1161/ATVBAHA.118.312314 31017822

[16] May F Hagedorn I Pleines I Bender M Vögtle T Eble J. CLEC-2 is an essential platelet-activating receptor in hemostasis and thrombosis. Blood. (2009) 114:3464–72. 10.1182/blood-2009-05-222273 19641185

[17] Suzuki-Inoue K Inoue O Ding G Nishimura S Hokamura K Eto K. Essential *in vivo* roles of the C-type lectin receptor CLEC-2: embryonic/neonatal lethality of CLEC-2-deficient mice by blood/lymphatic misconnections and impaired thrombus formation of CLEC-2-deficient platelets. J Biol Chem. (2010) 285:24494–507. 10.1074/jbc.M110.130575 20525685 PMC2915686

[18] Hughes CE Navarro-Núñez L Finney BA Mourão-Sá D Pollitt AY Watson SP CLEC-2 is not required for platelet aggregation at arteriolar shear. J Thromb Haemost. (2010) 8:2328–32. 10.1111/j.1538-7836.2010.04006.x 20695981 PMC4362701

[19] Ward LSC Sheriff L Marshall JL Manning JE Brill A Nash GB. Podoplanin regulates the migration of mesenchymal stromal cells and their interaction with platelets. J Cell Sci. (2019) 132:jcs222067. 10.1242/jcs.222067 30745334 PMC6432720

[20] Petri B Broermann A Li H Khandoga AG Zarbock A Krombach F. von Willebrand factor promotes leukocyte extravasation. Blood. (2010) 116:4712–9. 10.1182/blood-2010-03-276311 20716766

[21] Hara T Shimizu K Ogawa F Yanaba K Iwata Y Muroi E. Platelets control leukocyte recruitment in a murine model of cutaneous arthus reaction. Am J Pathol. (2010) 176:259–69. 10.2353/ajpath.2010.081117 20008131 PMC2797888

[22] Hidalgo A Chang J Jang JE Peired AJ Chiang EY Frenette PS. Heterotypic interactions enabled by polarized neutrophil microdomains mediate thromboinflammatory injury. Nat Med. (2009) 15:384–91. 10.1038/nm.1939 19305412 PMC2772164

[23] Kuligowski MP Kitching AR Hickey MJ. Leukocyte recruitment to the inflamed glomerulus: a critical role for platelet-derived P-selectin in the absence of rolling. J Immunol. (2006) 176:6991–9. 10.4049/jimmunol.176.11.6991 16709860

[24] Devi S Kuligowski MP Kwan RY Westein E Jackson SP Kitching AR. Platelet recruitment to the inflamed glomerulus occurs via an alphaIIbbeta3/GPVI-dependent pathway. Am J Pathol. (2010) 177:1131–42. 10.2353/ajpath.2010.091143 20651232 PMC2928948

[25] Schulz C von Brühl ML Barocke V Cullen P Mayer K Okrojek R. EMMPRIN (CD147/basigin) mediates platelet-monocyte interactions *in vivo* and augments monocyte recruitment to the vascular wall. J Thromb Haemost. (2011) 9:1007–19. 10.1111/j.1538-7836.2011.04235.x 21320284

[26] Pamuk GE Nuri Pamuk O Orüm H Arican O Turgut B Demir M. Elevated platelet-monocyte complexes in patients with psoriatic arthritis. Platelets. (2009) 20:493–7. 10.3109/09537100903165174 19852688

[27] Nicu EA Van der Velden U Nieuwland R Everts V Loos BG. Elevated platelet and leukocyte response to oral bacteria in periodontitis. J Thromb Haemost. (2009) 7:162–70. 10.1111/j.1538-7836.2008.03219.x 18983491

[28] Bunescu A Seideman P Lenkei R Levin K Egberg N. Enhanced Fcgamma receptor I, alphaMbeta2 integrin receptor expression by monocytes and neutrophils in rheumatoid arthritis: interaction with platelets. J Rheumatol. (2004) 31:2347–55. 15570633

[29] Dole VS Bergmeier W Mitchell HA Eichenberger SC Wagner DD. Activated platelets induce Weibel-Palade-body secretion and leukocyte rolling *in vivo*: role of P-selectin. Blood. (2005) 106:2334–9. 10.1182/blood-2005-04-1530 15956287 PMC1895274

[30] Nachman RL Rafii S. Platelets, petechiae, and preservation of the vascular wall. N Engl J Med. (2008) 359:1261–70. 10.1056/NEJMra0800887 18799560 PMC2935201

[31] Cloutier N Paré A Farndale RW Schumacher HR Nigrovic PA Lacroix S. Platelets can enhance vascular permeability. Blood. (2012) 120:1334–43. 10.1182/blood-2012-02-413047 22544703

[32] Moon DG van der Zee H Weston LK Gudewicz PW Fenton JW Kaplan JE. Platelet modulation of neutrophil superoxide anion production. Thromb Haemost. (1990) 63:91–6. 10.1055/s-0038-1645693 2160133

[33] Bazzoni G Dejana E Del Maschio A. Platelet-neutrophil interactions. Possible relevance in the pathogenesis of thrombosis and inflammation. Haematologica. (1991) 76:491–9. 1820986

[34] Clark SR Ma AC Tavener SA McDonald B Goodarzi Z Kelly MM. Platelet TLR4 activates neutrophil extracellular traps to ensnare bacteria in septic blood. Nat Med. (2007) 13:463–9. 10.1038/nm1565 17384648

[35] Kim S-J Jenne CN. Role of platelets in neutrophil extracellular trap (NET) production and tissue injury. Semin Immunol. (2016) 28:546–54. 10.1016/j.smim.2016.10.013 27876233

[36] Vivekananthan DP Bhatt DL Chew DP Zidar FJ Chan AW Moliterno DJ. Effect of clopidogrel pretreatment on periprocedural rise in C-reactive protein after percutaneous coronary intervention. Am J Cardiol. (2004) 94:358–60. 10.1016/j.amjcard.2004.04.035 15276105

[37] Nidorf SM Eikelboom JW Budgeon CA Thompson PL. Low-dose colchicine for secondary prevention of cardiovascular disease. J Am Coll Cardiol. (2013) 61:404–10. 10.1016/j.jacc.2012.10.027 23265346

[38] Deftereos S Giannopoulos G Efremidis M Kossyvakis C Katsivas A Panagopoulou V. Colchicine for prevention of atrial fibrillation recurrence after pulmonary vein isolation: mid-term efficacy and effect on quality of life. Heart Rhythm. (2014) 11:620–8. 10.1016/j.hrthm.2014.02.002 24508207

[39] Frostegård J. Immunity, atherosclerosis and cardiovascular disease. BMC Med. (2013) 11:117. 10.1186/1741-7015-11-117 23635324 PMC3658954

[40] Ross R Glomset J Harker L. Response to injury and atherogenesis. Am J Pathol. (1977) 86:675–84. 842616 PMC2032127

[41] Glagov S Hemodynamic Risk Factors: Mechanical Stress, Mural Architecture, Medial Nutrition and the Vulnerability of Arteries to Atherosclerosis (1972).

[42] Franck G Even G Gautier A Salinas M Loste A Procopio E. Haemodynamic stress-induced breaches of the arterial intima trigger inflammation and drive atherogenesis. Eur Heart J. (2019) 40:928–37. 10.1093/eurheartj/ehy822 30541066

[43] Massberg S Brand K Grüner S Page S Müller E Müller I. A critical role of platelet adhesion in the initiation of atherosclerotic lesion formation. J Exp Med. (2002) 196:887–96. 10.1084/jem.20012044 12370251 PMC2194025

[44] Fujimura Y Titani K Holland LZ Russell SR Roberts JR Elder JH. von Willebrand Factor a reduced and alkylated 52/48-kDa fragment beginning at amino acid residue 449 contains the domain interacting with platelet glycoprotein Ib. J Biol Chem. (1986) 261:381–5. 2934387

[45] Romo GM Dong JF Schade AJ Gardiner EE Kansas GS Li CQ. The glycoprotein Ib-IX-V complex is a platelet counterreceptor for P-selectin. J Exp Med. (1999) 190:803–14. 10.1084/jem.190.6.803 10499919 PMC2195629

[46] Ruggeri ZM Zarpellon A Roberts JR Mc Clintock RA Jing H Mendolicchio GL. Unravelling the mechanism and significance of thrombin binding to platelet glycoprotein Ib. Thromb Haemost. (2010) 104:894–902. 10.1160/TH10-09-0578 20941453 PMC3810535

[47] Simon DI Chen Z Xu H Li CQ Dong JF McIntire LV Platelet glycoprotein Ib is a counterreceptor for the leukocyte integrin Mac-1 (CD11b/CD18). J Exp Med. (2000) 192:193–204. 10.1084/jem.192.2.193 10899906 PMC2193258

[48] Koltsova E Sundd P Zarpellon A Ouyang H Mikulski Z Zampolli A Genetic deletion of platelet glycoprotein Ib alpha but not its extracellular domain protects from atherosclerosis. Thromb Haemost. (2014) 112:1252–63. 10.1160/th14-02-0130 25104056 PMC4429870

[49] Methia N André P Denis CV Economopoulos M Wagner DD. Localized reduction of atherosclerosis in von Willebrand factor-deficient mice. Blood. (2001) 98:1424–8. 10.1182/blood.V98.5.1424 11520791

[50] Ghasemzadeh M Kaplan ZS Alwis I Schoenwaelder SM Ashworth KJ Westein E. The CXCR1/2 ligand NAP-2 promotes directed intravascular leukocyte migration through platelet thrombi. Blood. (2013) 121:4555–66. 10.1182/blood-2012-09-459636 23550035 PMC3953078

[51] Wang Y Sakuma M Chen Z Ustinov V Shi C Croce K. Leukocyte engagement of platelet glycoprotein Ibα via the integrin Mac-1 is critical for the biological response to vascular injury. Circulation. (2005) 112:2993–3000. 10.1161/CIRCULATIONAHA.105.571315 16260637

[52] Strassel C Hechler B Bull A Gachet C Lanza F. Studies of mice lacking the GPIb-V-IX complex question the role of this receptor in atherosclerosis. J Thromb Haemost. (2009) 7:1935–8. 10.1111/j.1538-7836.2009.03608.x 19740100

[53] Massberg S Schürzinger K Lorenz M Konrad I Schulz C Plesnila N. Platelet adhesion via glycoprotein IIb integrin is critical for atheroprogression and focal cerebral ischemia: an *in vivo* study in mice lacking glycoprotein IIb. Circulation. (2005) 112:1180–8. 10.1161/CIRCULATIONAHA.105.539221 16103235

[54] Stalker TJ Newman DK Ma P Wannemacher KM Brass LF Platelet signaling. Handb Exp Pharmacol. (2012) 210:59–85. 10.1007/978-3-642-29423-5_3 PMC4785017 22918727

[55] Bültmann A Li Z Wagner S Peluso M Schönberger T Weis C. Impact of glycoprotein VI and platelet adhesion on atherosclerosis—A possible role of fibronectin. J Mol Cell Cardiol. (2010) 49:532–42. 10.1016/j.yjmcc.2010.04.009 20430036

[56] Fitzgerald DJ Roy L Catella F FitzGerald GA. Platelet activation in unstable coronary disease. N Engl J Med. (1986) 315:983–9. 10.1056/NEJM198610163151602 3531859

[57] Tschoepe D Roesen P Schwippert B Gries F. Platelets in diabetes: the role in the hemostatic regulation in atherosclerosis. Semin Thromb Hemostasis. (1993) 19:122–8. 10.1055/s-2007-994015 8356457

[58] Huo Y Schober A Forlow SB Smith DF Hyman MC Jung S. Circulating activated platelets exacerbate atherosclerosis in mice deficient in apolipoprotein E. Nat Med. (2003) 9:61–7. 10.1038/nm810 12483207

[59] Page C Pitchford S. Neutrophil and platelet complexes and their relevance to neutrophil recruitment and activation. Int Immunopharmacol. (2013) 17:1176–84. 10.1016/j.intimp.2013.06.004 23810443

[60] Dotsenko O Chaturvedi N Thom SA Wright AR Mayet J Shore A. Platelet and leukocyte activation, atherosclerosis and inflammation in European and South Asian men. J Thromb Haemost. (2007) 5:2036–42. 10.1111/j.1538-7836.2007.02711.x 17883700 PMC2650817

[61] Finsterbusch M Schrottmaier WC Kral-Pointner JB Salzmann M Assinger A. Measuring and interpreting platelet-leukocyte aggregates. Platelets. (2018) 29:677–85. 10.1080/09537104.2018.1430358 29461910 PMC6178087

[62] Tekelioglu Y Uzun H Güçer H. Circulating platelet-leukocyte aggregates in patients with inflammatory bowel disease. J Chin Med Assoc. (2013) 76:182–5. 10.1016/j.jcma.2012.12.015 23557884

[63] Burger PC Wagner DD. Platelet P-selectin facilitates atherosclerotic lesion development. Blood. (2003) 101:2661–6. 10.1182/blood-2002-07-2209 12480714

[64] Dong ZM Brown AA Wagner DD. Prominent role of P-selectin in the development of advanced atherosclerosis in ApoE-deficient mice. Circulation. (2000) 101:2290–5. 10.1161/01.CIR.101.19.2290 10811597

[65] Li D Wang Y Zhang L Luo X Li J Chen X. Roles of purinergic receptor P2Y, G protein-coupled 12 in the development of atherosclerosis in apolipoprotein E-deficient mice. Arterioscler Thromb Vasc Biol. (2012) 32:e81–9. 10.1161/ATVBAHA.111.239095 22628428

[66] Wihlborg AK Wang L Braun OO Eyjolfsson A Gustafsson R Gudbjartsson T. ADP receptor P2Y12 is expressed in vascular smooth muscle cells and stimulates contraction in human blood vessels. Arterioscler Thromb Vasc Biol. (2004) 24:1810–5. 10.1161/01.ATV.0000142376.30582.ed 15308557

[67] Boulaftali Y Owens AP Beale A Piatt R Casari C Lee RH. CalDAG-GEFI deficiency reduces atherosclerotic lesion development in mice. Arterioscler Thromb Vasc Biol. (2016) 36:792–9. 10.1161/ATVBAHA.115.306347 26988592 PMC4850098

[68] Afek A Kogan E Maysel-Auslender S Mor A Regev E Rubinstein A. Clopidogrel attenuates atheroma formation and induces a stable plaque phenotype in apolipoprotein E knockout mice. Microvasc Res. (2009) 77:364–9. 10.1016/j.mvr.2009.01.009 19323972

[69] Heim C Gebhardt J Ramsperger-Gleixner M Jacobi J Weyand M Ensminger SM. Clopidogrel significantly lowers the development of atherosclerosis in ApoE-deficient mice *in vivo*. Heart Vessels. (2016) 31:783–94. 10.1007/s00380-015-0696-7 26062773

[70] Schulz C Konrad I Sauer S Orschiedt L Koellnberger M Lorenz R. Effect of chronic treatment with acetylsalicylic acid and clopidogrel on atheroprogression and atherothrombosis in ApoE-deficient mice *in vivo*. Thromb Haemost. (2008) 99:190–5. 10.1160/TH07-03-0235 18217153

[71] Ganbaatar B Fukuda D Salim HM Nishimoto S Tanaka K Higashikuni Y. Ticagrelor, a P2Y12 antagonist, attenuates vascular dysfunction and inhibits atherogenesis in apolipoprotein-E-deficient mice. Atherosclerosis. (2018) 275:124–32. 10.1016/j.atherosclerosis.2018.05.053 29902700

[72] Xia X Li J Liang X Zhang S Liu T Liu J Ticagrelor suppresses oxidized low-density lipoprotein-induced endothelial cell apoptosis and alleviates atherosclerosis in ApoE-/- mice via downregulation of PCSK9. Mol Med Rep. (2018) 19:1453–62. 10.3892/mmr.2018.9779 30592271 PMC6390053

[73] Preusch MR Rusnak J Staudacher K Mogler C Uhlmann L Sievers P. Ticagrelor promotes atherosclerotic plaque stability in a mouse model of advanced atherosclerosis. Drug Des Dev Ther. (2016) 10:2691–9. 10.2147/DDDT.S105718 27616880 PMC5008449

[74] Hamilton JR Cornelissen I Mountford JK Coughlin SR *Atherosclerosis* proceeds independently of thrombin-induced platelet activation in ApoE^−/−^ mice. Atherosclerosis. (2009) 205:427–32. 10.1016/j.atherosclerosis.2009.01.018 19217621 PMC2717183

[75] Lutgens E Gorelik L Daemen MJ de Muinck ED Grewal IS Koteliansky VE. Requirement for CD154 in the progression of atherosclerosis. Nat Med. (1999) 5:1313–6. 10.1038/15271 10546000

[76] Sachais B Turrentine T Dawicki McKenna JM Rux AH Rader D Kowalska MA Elimination of platelet factor 4 (PF4) from platelets reduces atherosclerosis in C57Bl/6 and apoE^−/−^ mice. Thromb Haemost. (2007) 98:1108–13. 10.1160/TH07-04-0271 18000617

[77] Braunersreuther V Steffens S Arnaud C Pelli G Burger F Proudfoot A. A novel RANTES antagonist prevents progression of established atherosclerotic lesions in mice. Arterioscler Thromb Vasc Biol. (2008) 28:1090–6. 10.1161/ATVBAHA.108.165423 18388327

[78] Veillard NR Kwak B Pelli G Mulhaupt F James RW Proudfoot AE. Antagonism of RANTES receptors reduces atherosclerotic plaque formation in mice. Circ Res. (2004) 94:253–61. 10.1161/01.RES.0000109793.17591.4E 14656931

[79] Gerdes N Seijkens T Lievens D Kuijpers MJ Winkels H Projahn D. Platelet CD40 exacerbates atherosclerosis by transcellular activation of endothelial cells and leukocytes. Arterioscler Thromb Vasc Biol. (2016) 36:482–90. 10.1161/ATVBAHA.115.307074 26821950

[80] Cayatte AJ Du Y Oliver-Krasinski J Lavielle G Verbeuren TJ Cohen RA The thromboxane receptor antagonist S18886 but not aspirin inhibits atherogenesis in apo E-deficient mice: evidence that eicosanoids other than thromboxane contribute to atherosclerosis. Arterioscler Thromb Vasc Biol. (2000) 20:1724–8. 10.1161/01.ATV.20.7.1724 10894809

[81] Kobayashi T Tahara Y Matsumoto M Iguchi M Sano H Murayama T Roles of thromboxane A2 and prostacyclin in the development of atherosclerosis in apoE-deficient mice. J Clin Invest. (2004) 114:784–94. 10.1172/JCI21446 15372102 PMC516261

[82] McClelland S Gawaz M Kennerknecht E Konrad CS Sauer S Schuerzinger K. Contribution of cyclooxygenase-1 to thromboxane formation, platelet-vessel wall interactions and atherosclerosis in the ApoE null mouse. Atherosclerosis. (2009) 202:84–91. 10.1016/j.atherosclerosis.2008.04.016 18514659

[83] Cyrus T Sung S Zhao L Funk CD Tang S Praticò D. Effect of low-dose aspirin on vascular inflammation, plaque stability, and atherogenesis in low-density lipoprotein receptor-deficient mice. Circulation. (2002) 106:1282–7. 10.1161/01.CIR.0000027816.54430.96 12208806

[84] Kraus S Naumov I Shapira S Kazanov D Aroch I Afek A Aspirin but not meloxicam attenuates early atherosclerosis in apolipoprotein E knockout mice. Israel Med Assoc J. (2014) 16:233–8. 24834760

[85] Paul A Calleja L Camps J Osada J Vilella E Ferré N. The continuous administration of aspirin attenuates atherosclerosis in apolipoprotein E-deficient mice. Life Sci. (2000) 68:457–65. 10.1016/S0024-3205(00)00950-4 11205894

[86] Cyrus T Yao Y Tung LX Praticò D. Stabilization of advanced atherosclerosis in low-density lipoprotein receptor-deficient mice by aspirin. Atherosclerosis. (2006) 184:8–14. 10.1016/j.atherosclerosis.2004.10.047 16326168

[87] Tous M Ferré N Vilella E Riu F Camps J Joven J Aspirin attenuates the initiation but not the progression of atherosclerosis in apolipoprotein E-deficient mice fed a high-fat, high-cholesterol diet. Basic Clin Pharmacol Toxicol. (2004) 95:15–9. 10.1111/j.1742-7843.2004.pto950104.x 15245571

[88] Crittenden JR Bergmeier W Zhang Y Piffath CL Liang Y Wagner DD. CalDAG-GEFI integrates signaling for platelet aggregation and thrombus formation. Nat Med. (2004) 10:982–6. 10.1038/nm1098 15334074

[89] Stefanini L Roden RC Bergmeier W. CalDAG-GEFI is at the nexus of calcium-dependent platelet activation. Blood. (2009) 114:2506–14. 10.1182/blood-2009-04-218768 19628710

[90] Stefanini L Bergmeier W. CalDAG-GEFI and platelet activation. Platelets. (2010) 21:239–43. 10.3109/09537101003639931 20218908 PMC4119791

[91] Mann J Davies MJ. Mechanisms of progression in native coronary artery disease: role of healed plaque disruption. Heart. (1999) 82:265–8. 10.1136/hrt.82.3.265 10455072 PMC1729162

[92] Kuijpers MJ Gilio K Reitsma S Nergiz-Unal R Prinzen L Heeneman S. Complementary roles of platelets and coagulation in thrombus formation on plaques acutely ruptured by targeted ultrasound treatment: a novel intravital model. J Thromb Haemost. (2009) 7:152–61. 10.1111/j.1538-7836.2008.03186.x 18983512

[93] Hechler B Gachet C. Comparison of two murine models of thrombosis induced by atherosclerotic plaque injury. Thromb. Haemost. (2011) 105 (Suppl. 1):S3–12. 10.1160/THS10-11-0730 21479341

[94] Nergiz-Unal R Cosemans JM Feijge MA van der Meijden PE Storey RF van Giezen JJ. Stabilizing role of platelet P2Y(12) receptors in shear-dependent thrombus formation on ruptured plaques. PLoS ONE. (2010) 5:e10130. 10.1371/journal.pone.0010130 20405028 PMC2853564

[95] Feigin VL Norrving B Mensah GA. Global burden of stroke. Circ Res. (2017) 120:439–48. 10.1161/CIRCRESAHA.116.308413 28154096

[96] Mozaffarian D Benjamin EJ Go AS Arnett DK Blaha MJ Cushman M Executive Summary: Heart Disease and Stroke Statistics-−2016 Update. Circulation. (2016) 133:447–54. 10.1161/CIR.0000000000000366 26811276

[97] Gorelick PB Wong KS Bae H-J Pandey DK. Large artery intracranial occlusive disease: a large worldwide burden but a relatively neglected frontier. Stroke. (2008) 39:2396–9. 10.1161/STROKEAHA.107.505776 18535283

[98] Banerjee C Chimowitz MI. Stroke caused by atherosclerosis of the major intracranial arteries. Circ Res. (2017) 120:502–13. 10.1161/CIRCRESAHA.116.308441 28154100 PMC5312775

[99] NINDS Tissue plasminogen activator for acute ischemic stroke. N Engl J Med. (1995) 333:1581–7. 10.1056/NEJM199512143332401 7477192

[100] Broderick JP Berkhemer OA Palesch YY Dippel DW Foster LD Roos YB. Endovascular therapy is effective and safe for patients with severe ischemic stroke: pooled analysis of interventional management of stroke III and multicenter randomized clinical trial of endovascular therapy for acute ischemic stroke in the Netherlands data. Stroke. (2015) 46:3416–22. 10.1161/STROKEAHA.115.011397 26486865 PMC4659737

[101] Ducroux C Di Meglio L Loyau S Delbosc S Boisseau W Deschildre C Thrombus neutrophil extracellular traps content impair tPA-induced thrombolysis in acute ischemic stroke. Stroke. (2018) 49:754–7. 10.1161/STROKEAHA.117.019896 29438080

[102] Di Meglio L Desilles JP Mazighi M Ho-Tin-Noé B. Thrombolysis-resistant intracranial clot. Neurology. (2018) 90:1075. 10.1212/WNL.0000000000005645 29866933

[103] Rha J-H Saver JL. The impact of recanalization on ischemic stroke outcome: a meta-analysis. Stroke. (2007) 38:967–73. 10.1161/01.STR.0000258112.14918.24 17272772

[104] Alexandrov AV Grotta JC. Arterial reocclusion in stroke patients treated with intravenous tissue plasminogen activator. Neurology. (2002) 59:862–7. 10.1212/WNL.59.6.862 12297567

[105] Rubiera M Alvarez-Sabín J Ribo M Montaner J Santamarina E Arenillas JF. Predictors of early arterial reocclusion after tissue plasminogen activator-induced recanalization in acute ischemic stroke. Stroke. (2005) 36:1452–6. 10.1161/01.STR.0000170711.43405.81 15947260

[106] Saqqur M Molina CA Salam A Siddiqui M Ribo M Uchino K. Clinical deterioration after intravenous recombinant tissue plasminogen activator treatment: a multicenter transcranial doppler study. Stroke. (2007) 38:69–74. 10.1161/01.STR.0000251800.01964.f6 17138949

[107] Goerge T Ho-Tin-Noe B Carbo C Benarafa C Remold-O'Donnell E Zhao BQ. Inflammation induces hemorrhage in thrombocytopenia. Blood. (2008) 111:4958–64. 10.1182/blood-2007-11-123620 18256319 PMC2384127

[108] Kleinschnitz C Pozgajova M Pham M Bendszus M Nieswandt B Stoll G. Targeting platelets in acute experimental stroke: impact of glycoprotein Ib, VI, and IIb/IIIa blockade on infarct size, functional outcome, and intracranial bleeding. Circulation. (2007) 115:2323–30. 10.1161/CIRCULATIONAHA.107.691279 17438148

[109] Zhao BQ Chauhan AK Canault M Patten IS Yang JJ Dockal M. von Willebrand factor-cleaving protease ADAMTS13 reduces ischemic brain injury in experimental stroke. Blood. (2009) 114:3329–34. 10.1182/blood-2009-03-213264 19687510 PMC2759655

[110] Goebel S Li Z Vogelmann J Holthoff HP Degen H Hermann DM. The GPVI-Fc fusion protein revacept improves cerebral infarct volume and functional outcome in stroke. PLoS ONE. (2013) 8:e66960. 10.1371/journal.pone.0066960 23935828 PMC3720811

[111] Schuhmann MK Kraft P Bieber M Kollikowski AM Schulze H Nieswandt B Targeting platelet GPVI Plus rt-PA administration but not α2β1-mediated collagen binding protects against ischemic brain damage in mice. IJMS. (2019) 20:2019 10.3390/ijms20082019 PMC6515069 31022936

[112] Dumont B Lasne D Rothschild C Bouabdelli M Ollivier V Oudin C. Absence of collagen-induced platelet activation caused by compound heterozygous GPVI mutations. Blood. (2009) 114:1900–3. 10.1182/blood-2009-03-213504 19549989

[113] Stegner D Nieswandt B. Platelet receptor signaling in thrombus formation. J Mol Med. (2011) 89:109–21. 10.1007/s00109-010-0691-5 21058007

[114] Jiang P Jandrot-Perrus M. New advances in treating thrombotic diseases: GPVI as a platelet drug target. Drug Discov Today. (2014) 19:1471–5. 10.1016/j.drudis.2014.06.005 24931218

[115] Kraft P Schuhmann MK Fluri F Lorenz K Zernecke A Stoll G. Efficacy and safety of platelet glycoprotein receptor blockade in aged and comorbid mice with acute experimental stroke. Stroke. (2015) 46:3502–6. 10.1161/STROKEAHA.115.011114 26486866

[116] Vinik AI Erbas T Park TS Nolan R Pittenger GL. Platelet dysfunction in type 2 diabetes. Diabetes Care. (2001) 24:1476–85. 10.2337/diacare.24.8.1476 11473089

[117] Standley PR Ali S Bapna C Sowers JR. Increased platelet cytosolic calcium responses to low density lipoprotein in type II diabetes with and without hypertension. Am J Hypertens. (1993) 6:938–43. 10.1093/ajh/6.11.938 8305168

[118] Akai T Naka K Okuda K Takemura T Fujii S. Decreased sensitivity of platelets to prostacyclin in patients with diabetes mellitus. Horm Metab Res. (1983) 15:523–6. 10.1055/s-2007-1018778 6360841

[119] Grundy SM. Hypertriglyceridemia, atherogenic dyslipidemia, and the metabolic syndrome. Am J Cardiol. (1998) 81:18B−25B. 10.1016/S0002-9149(98)00033-2 9526809

[120] Tschoepe D Roesen P Kaufmann L Schauseil S Kehrel B Ostermann H. Evidence for abnormal platelet glycoprotein expression in diabetes mellitus. Eur J Clin Invest. (1990) 20:166–70. 10.1111/j.1365-2362.1990.tb02264.x 2112481

[121] Winocour PD Bryszewska M Watala C Rand ML Epand RM Kinlough-Rathbone RL. Reduced membrane fluidity in platelets from diabetic patients. Diabetes. (1990) 39:241–4. 10.2337/diab.39.2.241 2227132

[122] Striggow F Riek-Burchardt M Kiesel A Schmidt W Henrich-Noack P Breder J. Four different types of protease-activated receptors are widely expressed in the brain and are up-regulated in hippocampus by severe ischemia. Eur J Neurosci. (2001) 14:595–608. 10.1046/j.0953-816x.2001.01676.x 11556885

[123] Mao Y Zhang M Tuma RF Kunapuli SP. Deficiency of PAR4 attenuates cerebral ischemia/reperfusion injury in mice. J Cereb Blood Flow Metab. (2010) 30:1044–52. 10.1038/jcbfm.2009.283 20087365 PMC2949190

[124] Devanathan V Hagedorn I Köhler D Pexa K Cherpokova D Kraft P. Platelet Gi protein Gαi2 is an essential mediator of thrombo-inflammatory organ damage in mice. Proc Natl Acad Sci USA. (2015) 112:6491–6. 10.1073/pnas.1505887112 25944935 PMC4443332

[125] Schuhmann MK Kraft P Bieber M Haarmann A Homola GA Pham M. Influence of thrombolysis on the safety and efficacy of blocking platelet adhesion or secretory activity in acute ischemic stroke in mice. Transl Stroke Res. (2018) 9:493–8. 10.1007/s12975-017-0606-7 29322481

[126] Schuhmann MK Guthmann J Stoll G Nieswandt B Kraft P Kleinschnitz C. Blocking of platelet glycoprotein receptor Ib reduces thrombo-inflammation in mice with acute ischemic stroke. J Neuroinflamm. (2017) 14:18. 10.1186/s12974-017-0792-y 28109273 PMC5251224

[127] Uchiyama S Ishizuka N Shimada K Teramoto T Yamazaki T Oikawa S. Aspirin for stroke prevention in elderly patients with vascular risk factors: Japanese primary prevention project. Stroke. (2016) 47:1605–11. 10.1161/STROKEAHA.115.012461 27165949

[128] Gaziano JM Brotons C Coppolecchia R Cricelli C Darius H Gorelick PB. Use of aspirin to reduce risk of initial vascular events in patients at moderate risk of cardiovascular disease (ARRIVE): a randomised, double-blind, placebo-controlled trial. Lancet. (2018) 392:1036–46. 10.1016/S0140-6736(18)31924-X 30158069 PMC7255888

[129] McNeil JJ Wolfe R Woods RL Tonkin AM Donnan GA Nelson MR. Effect of aspirin on cardiovascular events and bleeding in the healthy elderly. N Engl J Med. (2018) 379:1509–18. 10.1056/NEJMoa1803955 30221597 PMC6289056

[130] Zinkstok SM Roos YB ARTIS Investigators. Early administration of aspirin in patients treated with alteplase for acute ischaemic stroke: a randomised controlled trial. Lancet. (2012) 380:731–7. 10.1016/S0140-6736(12)60949-0 22748820

[131] Zinkstok SM Beenen LF Majoie CB Marquering HA de Haan RJ Roos YB. Early deterioration after thrombolysis plus aspirin in acute stroke: a post hoc analysis of the antiplatelet therapy in combination with recombinant t-PA thrombolysis in ischemic stroke trial. Stroke. (2014) 45:3080–2. 10.1161/STROKEAHA.114.006268 25139873

[132] CAPRIE Steering Committee A randomised, blinded, trial of clopidogrel versus aspirin in patients at risk of ischaemic events (CAPRIE). Lancet. (1996) 348:1329–39. 10.1016/S0140-6736(96)09457-3 8918275

[133] Bhatt DL Fox KA Hacke W Berger PB Black HR Boden WE. Clopidogrel and aspirin versus aspirin alone for the prevention of atherothrombotic events. N Engl J Med. (2006) 354:1706–17. 10.1056/NEJMoa060989 16531616

[134] Diener HC Bogousslavsky J Brass LM Cimminiello C Csiba L Kaste M. Aspirin and clopidogrel compared with clopidogrel alone after recent ischaemic stroke or transient ischaemic attack in high-risk patients (MATCH): randomised, double-blind, placebo-controlled trial. Lancet. (2004) 364:331–7. 10.1016/S0140-6736(04)16721-4 15276392

[135] Wang Y Wang Y Zhao X Liu L Wang D Wang C Clopidogrel with aspirin in acute minor stroke or transient ischemic attack. N Engl J Med. (2013) 369:11–9. 10.1056/NEJMoa1215340 23803136

[136] Wang Y Pan Y Zhao X Li H Wang D Johnston SC. Clopidogrel with aspirin in acute minor stroke or transient ischemic attack (CHANCE) trial: one-year outcomes. Circulation. (2015) 132:40–6. 10.1161/CIRCULATIONAHA.114.014791 25957224

[137] Hong KS Lee SH Kim EG Cho KH Chang DI Rha JH. Recurrent ischemic lesions after acute atherothrombotic stroke: clopidogrel plus aspirin versus aspirin alone. Stroke. (2016) 47:2323–30. 10.1161/STROKEAHA.115.012293 27418597

[138] Johnston SC Easton JD Farrant M Barsan W Conwit RA Elm JJ. Clopidogrel and aspirin in acute ischemic stroke and high-risk TIA. N Engl J Med. (2018) 379:215–25. 10.1056/NEJMoa1800410 29766750 PMC6193486

[139] Tillman H Johnston SC Farrant M Barsan W Elm JJ Kim AS. Risk for major hemorrhages in patients receiving clopidogrel and aspirin compared with aspirin alone after transient ischemic attack or minor ischemic stroke: a secondary analysis of the POINT randomized clinical trial. JAMA Neurol. (2019) 76:774–82. 10.1001/jamaneurol.2019.0932 31034032 PMC6583063

[140] Johnston SC Amarenco P Albers GW Denison H Easton JD Evans SR Ticagrelor versus aspirin in acute stroke or transient ischemic attack. N Engl J Med. (2016) 375:35–43. 10.1056/NEJMoa1603060 27160892

[141] Amarenco P Albers GW Denison H Easton JD Evans SR Held P. Efficacy and safety of ticagrelor versus aspirin in acute stroke or transient ischaemic attack of atherosclerotic origin: a subgroup analysis of SOCRATES, a randomised, double-blind, controlled trial. Lancet Neurol. (2017) 16:301–10. 10.1016/S1474-4422(17)30038-8 28238711

[142] Wang Y Lin Y Meng X Chen W Chen G Wang Z. Effect of ticagrelor with clopidogrel on high on-treatment platelet reactivity in acute stroke or transient ischemic attack (PRINCE) trial: rationale and design. Int J Stroke. (2017) 12:321–5. 10.1177/1747493017694390 28381198

[143] Wang Y Johnston SC Bath PM Grotta JC Pan Y Amarenco P Acute dual antiplatelet therapy for minor ischaemic stroke or transient ischaemic attack. BMJ. (2019) 364:l895 10.1136/bmj.l895 30819687 PMC6394376

[144] Bath PM Woodhouse LJ Appleton JP Beridze M Christensen H Dineen RA. Antiplatelet therapy with aspirin, clopidogrel, and dipyridamole versus clopidogrel alone or aspirin and dipyridamole in patients with acute cerebral ischaemia (TARDIS): a randomised, open-label, phase 3 superiority trial. Lancet. (2018) 391:850–9. 10.1016/S0140-6736(17)32849-0 29274727 PMC5854459

[145] Adams HP Effron MB Torner J Dávalos A Frayne J Teal P. Emergency administration of abciximab for treatment of patients with acute ischemic stroke: results of an international phase III trial: abciximab in emergency treatment of stroke trial (AbESTT-II). Stroke. (2008) 39:87–99. 10.1161/STROKEAHA.106.476648 18032739

[146] Siebler M Hennerici MG Schneider D von Reutern GM Seitz RJ Röther J. Safety of tirofiban in acute ischemic stroke: the SaTIS trial. Stroke. (2011) 42:2388–92. 10.1161/STROKEAHA.110.599662 21852609

[147] Li L Geraghty OC Mehta Z Rothwell PM. Age-specific risks, severity, time course, and outcome of bleeding on long-term antiplatelet treatment after vascular events: a population-based cohort study. Lancet. (2017) 390:490–9. 10.1016/S0140-6736(17)30770-5 28622955 PMC5537194

[148] Antithrombotic Trialists (ATT) Collaboration Baigent C Blackwell L Collins R Emberson J Godwin J. Aspirin in the primary and secondary prevention of vascular disease: collaborative meta-analysis of individual participant data from randomised trials. Lancet. (2009) 373:1849–60. 10.1016/S0140-6736(09)60503-1 19482214 PMC2715005

[149] Antithrombotic Trialists (ATT) Collaboration Collaborative meta-analysis of randomised trials of antiplatelet therapy for prevention of death, myocardial infaction, and stroke in high risk patients. BMJ. (2002) 324:71–86. 10.1136/bmj.324.7329.71 11786451 PMC64503

[150] Gorelick PB Weisman SM. Risk of hemorrhagic stroke with aspirin use: an update. Stroke. (2005) 36:1801–7. 10.1161/01.STR.0000174189.81153.85 16020759

[151] Sandercock PA Counsell C Tseng M-C Cecconi E Oral antiplatelet therapy for acute ischaemic stroke. Cochrane Database Syst Rev. (2014) 26:CD000029 10.1002/14651858.CD000029.pub3 PMC6669270 24668137

[152] Powers WJ Rabinstein AA Ackerson T Adeoye OM Bambakidis NC Becker K. 2018 Guidelines for the early management of patients with acute ischemic stroke: a guideline for healthcare professionals from the American Heart Association/American *Stroke* Association. Stroke. (2018) 49:e46–99. 10.1161/STR.0000000000000172 29367334

[153] Reed GW Rossi JE Cannon CP. Acute myocardial infarction. Lancet. (2017) 389:197–210. 10.1016/S0140-6736(16)30677-8 27502078

[154] Yeh RW Sidney S Chandra M Sorel M Selby JV Go AS. Population trends in the incidence and outcomes of acute myocardial infarction. N Engl J Med. (2010) 362:2155–65. 10.1056/NEJMoa0908610 20558366

[155] Roffi M Patrono C Collet JP Mueller C Valgimigli M Andreotti F. 2015 ESC Guidelines for the management of acute coronary syndromes in patients presenting without persistent ST-segment elevation: Task Force for the Management of Acute Coronary Syndromes in Patients Presenting without Persistent ST-Segment Elevation of the European Society of Cardiology (ESC). Eur Heart J. (2016) 37:267–315. 10.1093/eurheartj/ehv320 26320110

[156] Ibanez B James S Agewall S Antunes MJ Bucciarelli-Ducci C Bueno H. 2017 ESC Guidelines for the management of acute myocardial infarction in patients presenting with ST-segment elevation. Eur Heart J. (2018) 39:119–77. 10.5603/KP.2018.0041 28886621

[157] Ndrepepa G Colleran R Kastrati A. Reperfusion injury in ST-segment elevation myocardial infarction: the final frontier. Coronary Artery Dis. (2017) 28:253–62. 10.1097/MCA.0000000000000468 28072597

[158] Amraotkar AR Song DD Otero D Trainor PJ Ismail I Kothari V. Platelet count and mean platelet volume at the time of and after acute myocardial infarction. Clin Appl Thromb/Hemostasis. (2017) 23:1052–9. 10.1177/1076029616683804 28294633 PMC5572529

[159] Yaghoubi A Golmohamadi Z Alizadehasl A Azarfarin R. Role of platelet parameters and haematological indices in myocardial infarction and unstable angina. J Pak Med Assoc. (2013) 63:5. 24601192

[160] van der Zee PM Biró E Ko Y de RJ Hack CE Sturk A P-selectin- and CD63-exposing platelet microparticles reflect platelet activation in peripheral arterial disease and myocardial infarction. Clin Chem. (2006) 52:657–64. 10.1373/clinchem.2005.057414 16439610

[161] Goto S Sakai H Goto M Ono M Ikeda Y Handa S. Enhanced shear-induced platelet aggregation in acute myocardial infarction. Circulation. (1999) 99:608–13. 10.1161/01.CIR.99.5.608 9950656

[162] Mauler M Herr N Schoenichen C Witsch T Marchini T Härdtner C. Platelet serotonin aggravates myocardial ischemia/reperfusion injury via neutrophil degranulation. Circulation. (2019) 139:918–31. 10.1161/CIRCULATIONAHA.118.033942 30586717 PMC6370531

[163] Furman MI Barnard MR Krueger LA Fox ML Shilale EA Lessard DM. Circulating monocyte-platelet aggregates are an early marker of acute myocardial infarction. J Am Coll Cardiol. (2001) 38:1002–6. 10.1016/S0735-1097(01)01485-1 11583872

[164] Ren F Mu N Zhang X Tan J Li L Zhang C. Increased platelet-leukocyte aggregates are associated with myocardial no-reflow in patients with ST elevation myocardial infarction. Am J Med Sci. (2016) 352:261–6. 10.1016/j.amjms.2016.05.034 27650230

[165] Du XJ Shan L Gao XM Kiriazis H Liu Y Lobo A. Role of intramural platelet thrombus in the pathogenesis of wall rupture and intra-ventricular thrombosis following acute myocardial infarction. Thromb Haemost. (2011) 105:356–64. 10.1160/TH10-07-0449 21057698

[166] Liu Y Gao XM Fang L Jennings NL Su Y Q X Novel role of platelets in mediating inflammatory responses and ventricular rupture or remodeling following myocardial infarction. Arterioscler Thromb Vasc Biol. (2011) 31:834–41. 10.1161/ATVBAHA.110.220467 21252067

[167] Pachel C Mathes D Arias-Loza AP Heitzmann W Nordbeck P Deppermann C. Inhibition of platelet GPVI protects against myocardial ischemia-reperfusion injury. Arterioscler Thromb Vasc Biol. (2016) 36:629–35. 10.1161/ATVBAHA.115.305873 26916731

[168] Takaya N Katoh Y Iwabuchi K Hayashi I Konishi H Itoh S. Platelets activated by collagen through the immunoreceptor tyrosine-based activation motif in the Fc receptor γ-chain play a pivotal role in the development of myocardial ischemia-reperfusion injury. J Mol Cell Cardiol. (2005) 39:856–64. 10.1016/j.yjmcc.2005.07.006 16246361

[169] Schönberger T Ziegler M Borst O Konrad I Nieswandt B Massberg S. The dimeric platelet collagen receptor GPVI-Fc reduces platelet adhesion to activated endothelium and preserves myocardial function after transient ischemia in mice. Am J Physiol Cell Physiol. (2012) 303:C757–66. 10.1152/ajpcell.00060.2012 22814400

[170] Vélez P Ocaranza-Sánchez R López-Otero D Grigorian-Shamagian L Rosa I Guitián E. Alteration of platelet GPVI signaling in ST-elevation myocardial infarction patients demonstrated by a combination of proteomic, biochemical, and functional approaches. Sci Rep. (2016) 6:39603. 10.1038/srep39603 28004756 PMC5177944

[171] Bigalke B Haap M Stellos K Geisler T Seizer P Kremmer E. Platelet glycoprotein VI (GPVI) for early identification of acute coronary syndrome in patients with chest pain. Thromb Res. (2010) 125:e184–9. 10.1016/j.thromres.2010.01.005 20122713

[172] Chien PT-Y Hsieh H-L Chi P-L Yang C-M PAR1-dependent COX-2/PGE_2_ production contributes to cell proliferation via EP_2_ receptors in primary human cardiomyocytes: thrombin induces COX-2 expression. Br J Pharmacol. (2014) 171:4504–19. 10.1111/bph.12794 24902855 PMC4209155

[173] Sabri A Guo J Elouardighi H Darrow AL Andrade-Gordon P Steinberg SF. Mechanisms of protease-activated receptor-4 Actions in cardiomyocytes: role of Src tyrosine kinase. J Biol Chem. (2003) 278:11714–20. 10.1074/jbc.M213091200 12522105

[174] Pawlinski R Tencati M Hampton CR Shishido T Bullard TA Casey LM. Protease-activated receptor-1 contributes to cardiac remodeling and hypertrophy. Circulation. (2007) 116:2298–306. 10.1161/CIRCULATIONAHA.107.692764 17967980 PMC2848478

[175] Strande JL Hsu A Su J Fu X Gross GJ Baker JE. SCH 79797, a selective PAR1 antagonist, limits myocardial ischemia/reperfusion injury in rat hearts. Basic Res Cardiol. (2007) 102:350–8. 10.1007/s00395-007-0653-4 17468933 PMC3942648

[176] Antoniak S Pawlinski R Mackman N. Protease-activated receptors and myocardial infarction. IUBMB Life. (2011) 63:383–9. 10.1002/iub.441 21438116 PMC3121912

[177] Kolpakov MA Rafiq K Guo X Hooshdaran B Wang T Vlasenko L. Protease-activated receptor 4 deficiency offers cardioprotection after acute ischemia reperfusion injury. J Mol Cell Cardiol. (2016) 90:21–9. 10.1016/j.yjmcc.2015.11.030 26643815 PMC5332160

[178] Strande JL Hsu A Su J Fu X Gross GJ Baker JE. Inhibiting protease-activated receptor 4 limits myocardial ischemia/reperfusion injury in rat hearts by unmasking adenosine signaling. J Pharmacol Exp Therapeut. (2008) 324:1045–54. 10.1124/jpet.107.133595 18055876 PMC2935083

[179] Barrabés JA Inserte J Mirabet M Quiroga A Hernando V Figueras J. Antagonism of P2Y12 or GPIIb/IIIa receptors reduces platelet-mediated myocardial injury after ischaemia and reperfusion in isolated rat hearts. Thromb Haemost. (2010) 104:128–35. 10.1160/TH09-07-0440 20431845

[180] Adamek A Hu K Bayer B Wagner H Ertl G Bauersachs J. High dose aspirin and left ventricular remodeling after myocardial infarction: aspirin and myocardial infarction. Basic Res Cardiol. (2007) 102:334–40. 10.1007/s00395-007-0647-2 17340057

[181] Xu XR Carrim N Neves MA McKeown T Stratton TW Coelho RM. Platelets and platelet adhesion molecules: novel mechanisms of thrombosis and anti-thrombotic therapies. Thromb J. (2016) 14:29. 10.1186/s12959-016-0100-6 27766055 PMC5056500

[182] Hohlfeld T Braun M Strobach H Schrör K. Protection of reperfused ischemic pig myocardium by nexopamil, a new combined Ca^2+^ and serotonin antagonist. J Cardiovasc Pharmacol. (1994) 23:922–31. 10.1097/00005344-199406000-00010 7523784

[183] Oostingh GJ Pozgajova M Ludwig RJ Krahn T Boehncke WH Nieswandt B. Diminished thrombus formation and alleviation of myocardial infarction and reperfusion injury through antibody- or small-molecule-mediated inhibition of selectin-dependent platelet functions. Haematologica. (2007) 92:502–12. 10.3324/haematol.10741 17488661

[184] The CURE Trial Investigators Effects of clopidogrel in addition to aspirin in patients with acute coronary syndromes without ST segment elevation. BMJ. (2007) 334:1265–9. 10.1136/bmj.39220.618646.AE 17569934 PMC1892517

[185] Wiviott SD Braunwald E McCabe CH Montalescot G Ruzyllo W Gottlieb S. Prasugrel versus clopidogrel in patients with acute coronary syndromes. N Engl J Med. (2007) 357:2001–15. 10.1056/NEJMoa0706482 17982182

[186] Wallentin L Becker RC Budaj A Cannon CP Emanuelsson H Held C. Ticagrelor versus clopidogrel in patients with acute coronary syndromes. N Engl J Med. (2009) 361:1045–57. 10.1056/NEJMoa0904327 19717846

[187] Bonaca MP Bhatt DL Cohen M Steg PG Storey RF Jensen EC. Long-term use of ticagrelor in patients with prior myocardial infarction. N Engl J Med. (2015) 372:1791–800. 10.1056/NEJMoa1500857 25773268

[188] Bonaca MP Wiviott SD Morrow DA Steg PG Hamm C Bhatt DL. Reduction in subtypes and sizes of myocardial infarction with ticagrelor in PEGASUS-TIMI 54. J Am Heart Assoc. (2018) 7:e009260. 10.1161/JAHA.118.009260 30571502 PMC6404436

[189] Bonaca MP Storey RF Theroux P Steg PG Bhatt DL Cohen MC. Efficacy and safety of ticagrelor over time in patients with prior MI in PEGASUS-TIMI 54. J Am Coll Cardiol. (2017) 70:1368–75. 10.1016/j.jacc.2017.07.768 28882235

[190] Morrow DA Braunwald E Bonaca MP Ameriso SF Dalby AJ Fish MP. Vorapaxar in the secondary prevention of atherothrombotic events. N Engl J Med. (2012) 366:1404–13. 10.1056/NEJMoa1200933 22443427

[191] Leonardi S Tricoci P White HD Armstrong PW Huang Z Wallentin L. Effect of vorapaxar on myocardial infarction in the thrombin receptor antagonist for clinical event reduction in acute coronary syndrome (TRA.CER) trial. Eur Heart J. (2013) 34:1723–31. 10.1093/eurheartj/eht104 23530022

[192] Eckstein HH Böckler D Flessenkämper I Schmitz-Rixen T Debus S Lang W. Ultrasonographic screening for the detection of abdominal aortic aneurysms. Dtsch Arztebl Int. (2009) 106:657–63. 10.3238/arztebl.2009.0657 19946430 PMC2780009

[193] Wang Y-D Liu Z-J Ren J Xiang M-X. Pharmacological therapy of abdominal aortic aneurysm: an update. Curr Vasc Pharmacol. (2018) 16:114–24. 10.2174/1570161115666170413145705 28412911

[194] Cameron SJ Russell HM Owens AP Antithrombotic therapy in abdominal aortic aneurysm: beneficial or detrimental? Blood. (2018) 132:2619–28. 10.1182/blood-2017-08-743237 30228233 PMC6302498

[195] Kazi M Thyberg J Religa P Roy J Eriksson P Hedin U. Influence of intraluminal thrombus on structural and cellular composition of abdominal aortic aneurysm wall. J Vasc Surg. (2003) 38:1283–92. 10.1016/S0741-5214(03)00791-2 14681629

[196] Behr-Rasmussen C Grøndal N Bramsen MB Thomsen MD Lindholt JS. Mural thrombus and the progression of abdominal aortic aneurysms: a large population-based prospective cohort study. Eur J Vasc Endovasc Surg. (2014) 48:301–7. 10.1016/j.ejvs.2014.05.014 24969094

[197] Touat Z Ollivier V Dai J Huisse MG Bezeaud A Sebbag U. Renewal of mural thrombus releases plasma markers and is involved in aortic abdominal aneurysm evolution. Am J Pathol. (2006) 168:1022–30. 10.2353/ajpath.2006.050868 16507915 PMC1606522

[198] Delbosc S Alsac JM Journe C Louedec L Castier Y Bonnaure-Mallet M. *Porphyromonas gingivalis* participates in pathogenesis of human abdominal aortic aneurysm by neutrophil activation. Proof of concept in rats. PLoS ONE. (2011) 6:e18679. 10.1371/journal.pone.0018679 21533243 PMC3076426

[199] Adolph R Vorp DA Steed DL Webster MW Kameneva MV Watkins SC. Cellular content and permeability of intraluminal thrombus in abdominal aortic aneurysm. J Vasc Surg. (1997) 25:916–26. 10.1016/S0741-5214(97)70223-4 9152321

[200] Michel JB Martin-Ventura JL Egido J Sakalihasan N Treska V Lindholt J. Novel aspects of the pathogenesis of aneurysms of the abdominal aorta in humans. Cardiovasc Res. (2011) 90:18–27. 10.1093/cvr/cvq337 21037321 PMC3058728

[201] Khan JA Abdul Rahman MN Mazari FA Shahin Y Smith G Madden L. Intraluminal thrombus has a selective influence on matrix metalloproteinases and their inhibitors (tissue inhibitors of matrix metalloproteinases) in the wall of abdominal aortic aneurysms. Ann Vasc Surg. (2012) 26:322–9. 10.1016/j.avsg.2011.08.015 22305865

[202] Fontaine V Touat Z Mtairag el M Vranckx R Louedec L Houard X. Role of leukocyte elastase in preventing cellular re-colonization of the mural thrombus. Am J Pathol. (2004) 164:2077–87. 10.1016/S0002-9440(10)63766-2 15161642 PMC1615778

[203] Swedenborg J Eriksson P. The intraluminal thrombus as a source of proteolytic activity. Ann N Y Acad Sci. (2006) 1085:133–8. 10.1196/annals.1383.044 17182929

[204] Saraff K Babamusta F Cassis LA Daugherty A. Aortic dissection precedes formation of aneurysms and atherosclerosis in angiotensin II-infused, apolipoprotein E-deficient mice. Arterioscler Thromb Vasc Biol. (2003) 23:1621–6. 10.1161/01.ATV.0000085631.76095.64 12855482

[205] Anidjar S Salzmann JL Gentric D Lagneau P Camilleri JP Michel JB. Elastase-induced experimental aneurysms in rats. Circulation. (1990) 82:973–81. 10.1161/01.CIR.82.3.973 2144219

[206] Pyo R Lee JK Shipley JM Curci JA Mao D Ziporin SJ. Targeted gene disruption of matrix metalloproteinase-9 (gelatinase B) suppresses development of experimental abdominal aortic aneurysms. J Clin Invest. (2000) 105:1641–9. 10.1172/JCI8931 10841523 PMC300851

[207] Lu G Su G Davis JP Schaheen B Downs E Roy RJ. A novel chronic advanced stage abdominal aortic aneurysm murine model. J Vasc Surg. (2017) 66:232–42.e4. 10.1016/j.jvs.2016.07.105 28274752 PMC5483384

[208] Allaire E Muscatelli-Groux B Guinault AM Pages C Goussard A Mandet C. vascular smooth muscle cell endovascular therapy stabilizes already developed aneurysms in a model of aortic injury elicited by inflammation and proteolysis. Ann Surg. (2004) 239:417–27. 10.1097/01.sla.0000114131.79899.82 15075661 PMC1356242

[209] Dai J Louedec L Philippe M Michel JB Houard X. Effect of blocking platelet activation with AZD6140 on development of abdominal aortic aneurysm in a rat aneurysmal model. J Vasc Surg. (2009) 49:719–27. 10.1016/j.jvs.2008.09.057 19028049

[210] Liu O Jia L Liu X Wang Y Wang X Qin Y. Clopidogrel, a platelet P2Y12 receptor inhibitor, reduces vascular inflammation and angiotensin II induced-abdominal aortic aneurysm progression. PLoS ONE. (2012) 7:e51707. 10.1371/journal.pone.0051707 23284748 PMC3527447

[211] Owens AP Edwards TL Antoniak S Geddings JE Jahangir E Wei WQ. Platelet inhibitors reduce rupture in a mouse model of established abdominal aortic aneurysm. Arterioscler Thromb Vasc Biol. (2015) 35:2032–41. 10.1161/ATVBAHA.115.305537 26139462 PMC4552620

[212] Lindholt JS Sorensen HT Michel JB Thomsen HF Henneberg EW. Low-dose aspirin may prevent growth and later surgical repair of medium-sized abdominal aortic aneurysms. Vasc Endovasc Surg. (2008) 42:329–34. 10.1177/1538574408315205 18728038

[213] Wanhainen A Mani K Kullberg J Svensjö S Bersztel A Karlsson L The effect of ticagrelor on growth of small abdominal aortic aneurysms—a randomized controlled trial. Cardiovasc. Res. (2019) 2019:cvz133 10.1093/cvr/cvz133 31135888

